# Acaricide, Fungicide and Drug Interactions in Honey Bees (*Apis mellifera*)

**DOI:** 10.1371/journal.pone.0054092

**Published:** 2013-01-29

**Authors:** Reed M. Johnson, Lizette Dahlgren, Blair D. Siegfried, Marion D. Ellis

**Affiliations:** Department of Entomology, University of Nebraska – Lincoln, Lincoln, Nebraska, United States of America; Royal Holloway University of London, United Kingdom

## Abstract

**Background:**

Chemical analysis shows that honey bees (*Apis mellifera*) and hive products contain many pesticides derived from various sources. The most abundant pesticides are acaricides applied by beekeepers to control *Varroa destructor*. Beekeepers also apply antimicrobial drugs to control bacterial and microsporidial diseases. Fungicides may enter the hive when applied to nearby flowering crops. Acaricides, antimicrobial drugs and fungicides are not highly toxic to bees alone, but in combination there is potential for heightened toxicity due to interactive effects.

**Methodology/Principal Findings:**

Laboratory bioassays based on mortality rates in adult worker bees demonstrated interactive effects among acaricides, as well as between acaricides and antimicrobial drugs and between acaricides and fungicides. Toxicity of the acaricide tau-fluvalinate increased in combination with other acaricides and most other compounds tested (15 of 17) while amitraz toxicity was mostly unchanged (1 of 15). The sterol biosynthesis inhibiting (SBI) fungicide prochloraz elevated the toxicity of the acaricides tau-fluvalinate, coumaphos and fenpyroximate, likely through inhibition of detoxicative cytochrome P450 monooxygenase activity. Four other SBI fungicides increased the toxicity of tau-fluvalinate in a dose-dependent manner, although possible evidence of P450 induction was observed at the lowest fungicide doses. Non-transitive interactions between some acaricides were observed. Sublethal amitraz pre-treatment increased the toxicity of the three P450-detoxified acaricides, but amitraz toxicity was not changed by sublethal treatment with the same three acaricides. A two-fold change in the toxicity of tau-fluvalinate was observed between years, suggesting a possible change in the genetic composition of the bees tested.

**Conclusions/Significance:**

Interactions with acaricides in honey bees are similar to drug interactions in other animals in that P450-mediated detoxication appears to play an important role. Evidence of non-transivity, year-to-year variation and induction of detoxication enzymes indicates that pesticide interactions in bees may be as complex as drug interactions in mammals.

## Introduction

Chemical analysis of honey bees (*Apis mellifera*) and hive products show that most managed bee colonies in North America and Europe are repositories of a suite of chemical contaminants, including an assortment of insecticides, acaricides, herbicides and fungicides [1]–[3]. While a number of the residues detected were insecticides, many of which are highly toxic to bees, compounds of low acute toxicity were detected most frequently and at the highest concentrations in both bees and hive products. Some of the most ubiquitous contaminants of bees and bee products, coumaphos and tau-fluvalinate [3], are abundant in the hive environment because both are deliberately introduced as therapeutic acaricides to control the ectoparasitic mite, *Varroa destructor*. *Varroa* is the most serious pest of managed honey bee colonies in Europe and North America and clearly plays a role in the recent colony losses associated with colony collapse disorder [4]–[6]. *Varroa* weakens colonies in two ways: directly, by consuming the hemolymph of adult and pupal bees and, indirectly, by vectoring honey bee viruses and causing immunosuppression in parasitized bees [7].

In the face of the serious challenges presented by *Varroa,* beekeeping has become dependent on management techniques to control mite infestations, with apicultural acaricides playing a major role [4]. However, finding chemical control agents that selectively kill an arthropod pest of an arthropod host poses a unique pharmacological challenge. Synthetic pesticides that have been used as acaricides include the pyrethroid tau-fluvalinate (Apistan and Mavrik), the organophosphate coumaphos (CheckMite+, Perizin and Asuntol 50), the formamidine amitraz (Apivar and Taktik) and the pyrazole fenpyroximate (Hivastan and FujiMite). Natural products are also used for *Varroa* control, including the monoterpenoid thymol (ApilifeVar and ApiGuard) and the organic acids, oxalic acid (Oxivar) and formic acid (MiteAway Quick Strips). There is little doubt that bees can benefit from reduced *Varroa* populations through the effective use of acaricides in combination with other management techniques [4], [6], [7]. In a chemical survey of honey bee colonies suffering from colony collapse disorder, the healthiest colonies were found to have higher concentrations of one acaricide, coumaphos [8].

The effectiveness of tau-fluvalinate [9] and coumaphos [10] has waned as *Varroa* populations have developed resistance to these acaricides. However, tau-fluvalinate and coumaphos remain common contaminants in the hive environment, partially as a result of continued application by beekeepers, and partially due to their lipophilic properties which lead to accumulation and persistence in beeswax [1], [11]. Both coumaphos and tau-fluvalinate survive the wax recycling process and are present in newly manufactured wax foundation [3], [12]. While amitraz itself does not accumulate in bee colonies [13], the amitraz metabolite 2,4-dimethyl formamide (DPMF) has been detected in both bees and wax [3]. Oxalic acid is a natural product that can be found in honey and as an allelochemical in plants [14], though not at concentrations used for *Varroa* control. While thymol and other monoterpenoids may be naturally present in floral sources at low concentration, the high concentrations needed for acaricidal activity may noticeably contaminate honey and wax [12], [15]. With the wide range of acaricides currently in use and the continued presence of lipophilic acaricides in beeswax, it is quite likely that bees will be exposed to multiple acaricides simultaneously.

In addition to the acaricides, beekeepers may also apply antimicrobial drugs to control bacterial and microsporidial pathogens. Fumagillin (Fumadil-B) is fed in sucrose syrup to control infection by the microsporidian gut pathogens *Nosema apis* and *Nosema ceranae*. Oxytetracycline (Terramycin™) and tylosin (Tylan™) are applied in powdered sugar or syrup to control American foulbrood (*Paenibacillus larvae*) and other bacterial infections. To protect harvested honey from contamination many antimicrobial drugs and acaricides are subject to a withholding period during which these therapeutics cannot be applied. As such, beekeepers are left with a relatively narrow window during which antimicrobial or acaricide applications are possible, potentially leading to a situation where multiple treatments are applied simultaneously.

In addition to compounds applied by the beekeeper, bees may also be exposed to plant protection products applied to flowers and flowering crops. Fungicides are the most abundant and common of the plant protection products found in bees and bee products because fungicides can be applied during bloom when bees are present [3], [8], [16]. While fungicides generally appear safe for adult bees [17], these compounds may, in certain situations, produce harmful effects [18]. For instance chlorothalonil (Bravo), the most commonly detected fungicide in bees and bee products [3], was found in “entombed pollen” in colonies suffering from colony collapse disorder [19]. Larval and pupal mortality has been reported in bees exposed to the fungicides pyraclostrobin and boscalid, which together constitute Pristine [16]. There are also documented interactions between the sterol biosynthesis inhibiting (SBI) fungicides and pyrethroid insecticides in honey bees [20]–[22]. For example, prochloraz is a SBI fungicide that functions through the inhibition of fungal cytochrome P450-monooxygenase (P450) mediated synthesis of ergosterols. Prochloraz has been shown to inhibit detoxicative P450 activity in honey bees as well, particularly in relation to detoxication of pyrethroid pesticides [20], [21], [23], [24]. Pristine and a variety of SBI fungicides are used during bloom on almond orchards, when bees are present, and have been detected in pollen samples [3], [16].

With the potential for managed honey bees to experience simultaneous exposure to acaricides, antimicrobials and fungicides it is important to consider the potential for harmful interactions between these compounds. Any interactions observed could provide an insight into honey bee physiology and will shed light on bees’ mechanisms of tolerance for both natural and synthetic xenobiotics. The present study aims to test for interactions between these most abundant contaminants of the hive environment using pair-wise lethal dose bioassays in which a sublethal pre-treatment with one acaricide, fungicide or antimicrobial is followed by a series of lethal doses of an acaricide. Mortality counts were then used to fit log-probit regression lines and determine lethal dose values (LD_50_s). Acaricides were also combined with model enzyme inhibitors to characterize the classes of detoxicative enzymes that may be the basis for interactive effects.

Bliss [25], recognized three principal types of interactive effects that pesticide or drug combinations may elicit [26]. If no interaction occurs, and a combination is found to be only as deadly as its most toxic constituent, then the components of a mixture are understood to act independently. Such “independent joint action” is the null hypothesis for the experiments presented here and is characterized by acaricide toxicity that is unchanged by prior exposure to any other compound.

**Figure 1:**
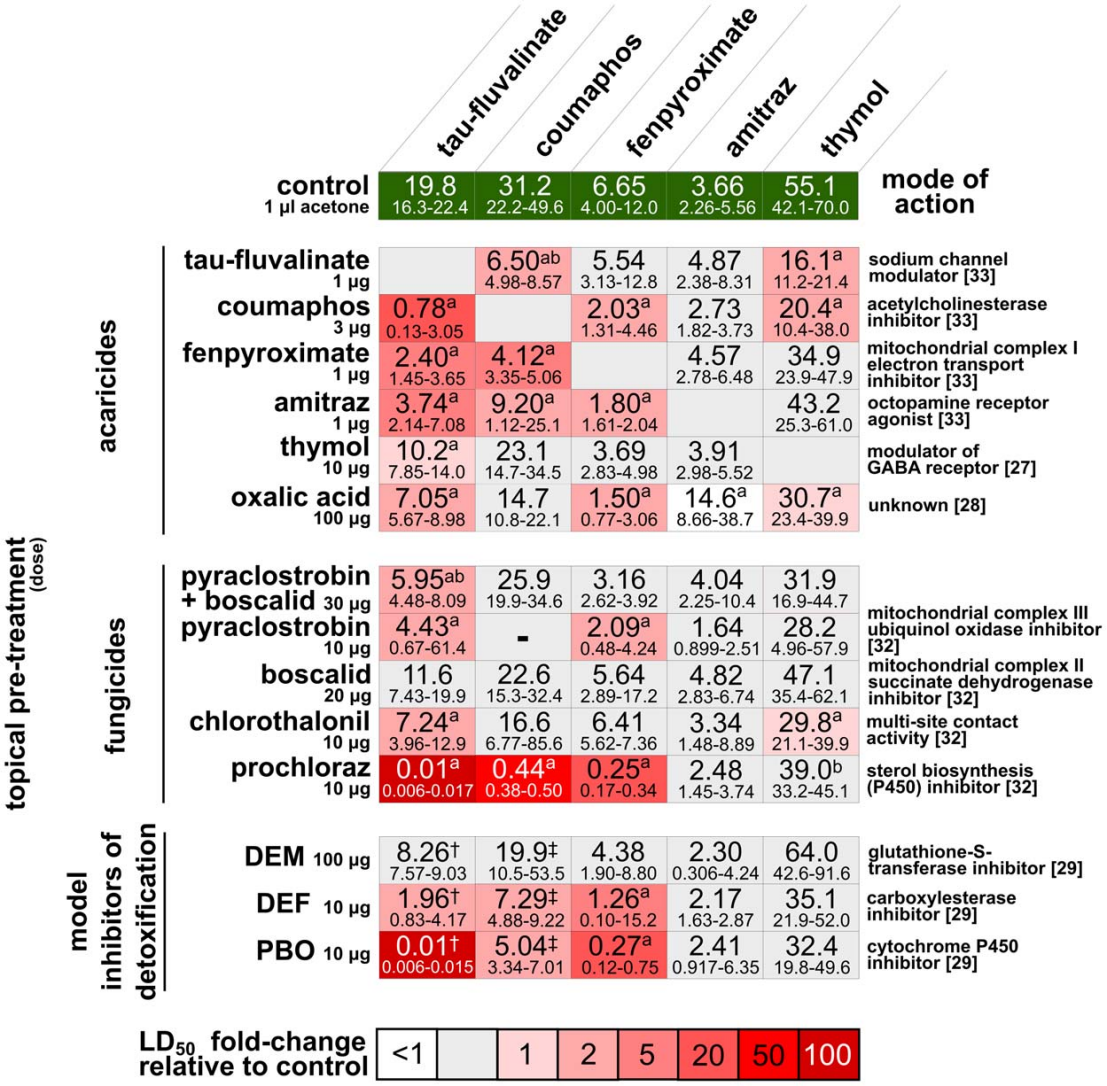
Median lethal dose (LD_50_) of acaricides to honey bees in 2009 following sublethal treatment with acaricides, fungicides, or detoxicative enzyme inhibitors. Confidence intervals (95%) are indicated below the LD_50_ values. Significant differences compared to the control treatment are indicated with a superscript letter: a = significant pre-treatment effect, b = significant pre-treatment*acaricide dose effect (Table S1). LD_50_ values taken from previous work: † = [35], ‡ = [22]. Names for classical enzyme inhibitors are abbreviated as follows DEM = diethyl maleate, DEF = S,S,S-tributylphosphorotrithioate, PBO = piperonyl butoxide. A dash “−” indicates an LD_50_ that could not be calculated because of insufficient data.

**Figure 2:**
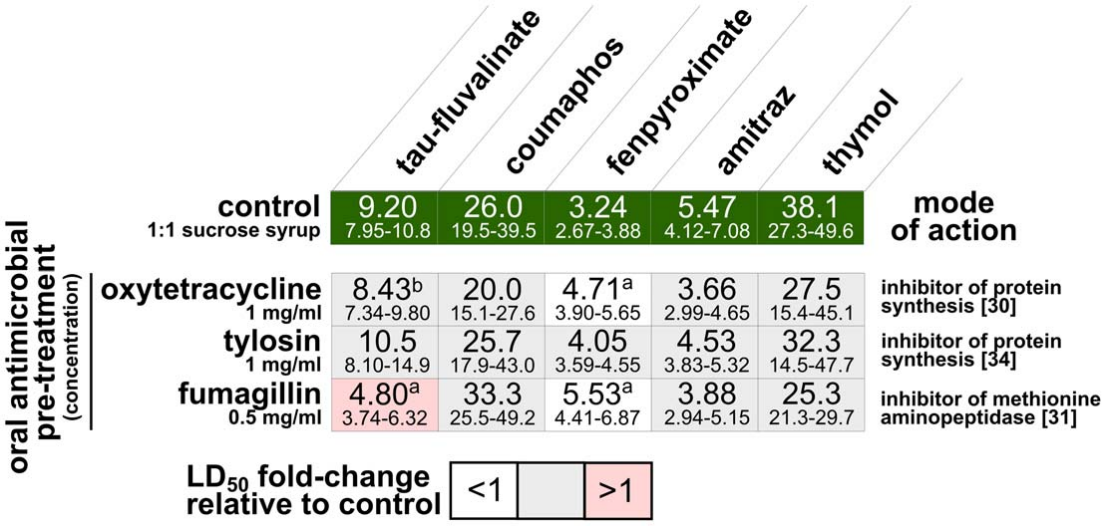
Median lethal doses (LD_50_) of acaricides to honey bees in 2010 fed antimicrobial drugs used in beekeeping. Confidence intervals (95%) are indicated below the LD_50_ values. Significant differences compared to the respective treatment are indicated with a superscript letter a = significant pre-treatment effect, b = significant pre-treatment*acaricide dose effect (Table S2).

Interactive effects between compounds are observed when the toxicity of a drug or pesticide combination is either more or less toxic than expected based on the toxicity of the most toxic constituent. In the context of these experiments an interactive effect is defined as a change in the toxicity of an acaricide following sublethal pre-treatment with a fungicide, antimicrobial drug or another acaricide. An agonistic interaction is defined by the elevated toxicity of a drug or pesticide combination, while an antagonistic interaction is characterized by decreased toxicity. Interpreting the biological basis of interactive effects between compounds requires that the mode of action of the drugs or pesticides are known. Additive agonistic interactions are most likely to occur when different compounds work through the same mode of action. Synergistic agonistic interactions probably occur when the compounds work through different modes of action. The mode of action for each compound is listed in Figure 1 and 2 for each acaricide, fungicide and antimicrobial compound [27]–[34].

## Materials and Methods

Bees were first treated with a sublethal dose of either an acaricide, a fungicide, an antimicrobial drug, or an enzyme inhibitor. Bees were then treated with a range of lethal doses of an acaricide to estimate the dose-response curve. Sublethal pre-treaments included: six acaricides (tau-fluvalinate, coumaphos, fenpyroximate, amitraz, thymol and oxalic acid); eight fungicides of which three were non-SBI (pyraclostrobin, boscalid, chlorothalonil) and five were SBI fungicides (prochloraz, propiconazole, fenbuconazole, metconazole and myclobutanil); three antimicrobials (oxytetracycline, tylosin and fumagillin); and three enzyme inhibitors (diethyl maleate, S,S,S-tributylphosphorotrithioate, and piperonyl butoxide). Lethal doses of five acaricides (i.e. all except oxalic acid) were applied as a second treatment. We tested 25 of the 6×6 possible acaricide-acaricide combinations, 24 of the 6×5 fungicide-acaricide combinations, 15 of the 6×3 antimicrobial-acaricide combinations and 9 of the 6×3 enzyme inhibitor-acaricide combinations. The acaricide oxalic acid was never used as a lethal agent in pairwise combinations because it was not possible to deliver a lethal dose of this compound in the standardized volume of solvent. Enzyme inhibitors were not tested with tau-fluvalinate or coumaphos as these combinations have been tested previously [22], [35]. The combination of pyraclostrobin and coumaphos was tested, but insufficient data were collected to allow for a meaningful comparison. The fungicides propiconazole, fenbuconazole, metconazole and myclobutanil were tested in combination with a single acaricide, tau-fluvalinate.

### Chemicals

Technical grade chemicals were used for all trials, with the exception of the antimicrobial drugs. The acaricides coumaphos, fenpyroximate and tau-fluvalinate were obtained from Chem Services Inc. (West Chester, PA), thymol from Aldrich Chemical Co. (Milwaukee, WI) and oxalic acid from Fisher Scientific (Rochester, NY). The model enzyme inhibitors piperonyl butoxide (PBO) and diethyl maleate (DEM) were obtained from Acros Organics (Morris Plains, NJ) and S,S,S-tributylphosphorotrithioate (DEF) from Chem Services. The fungicides boscalid, pyraclostrobin, prochloraz, fenbuconazole, metconazole, myclobutanil and propiconazole were also purchased from Chem Services, and chlorothalonil from Fluka Analytical (St. Louis, MO). All chemicals were serially diluted in HPLC-grade acetone for topical application.

### Insects

Fourteen honey bee colonies maintained at the University of Nebraska – Lincoln East Campus provided bees for bioassays conducted from April – September 2009 and 2010. Colonies were requeened in 2010 with naturally mated Italian queens (C. F. Koehnen & Sons, Inc., Glenn, CA). Bacterial brood diseases were prevented with Terramycin (oxytetracycline) treatments in March of both years. *Nosema* spp. infection was controlled using Fumagilin-B (fumagillin) fed in March 2009. Apiguard and oxalic acid were the only acaricides used in the apiary to control *Varroa* populations during the four years prior to conducting this study.

Frames of late-stage brood were collected from these colonies and placed in a dark, humid incubator (Darwin Chambers Co., St. Louis, MO, model H024) at 34°C. Newly emerged adults were brushed from frames daily into screened wooden cages (21×14×12 cm) and weighed as a group to estimate the number of bees. Each cohort of 200–800 bees was provisioned with 1∶1 (w/w) granulated sugar dissolved in water and held for 3–4 days in the incubator.

### Pre-treatment with Sublethal Doses of Acaricide, Fungicide, Antimicrobial Drug or Inhibitor

Each treatment series was conducted using 3–4 day-old adult worker bees divided into 8–13 subgroups of 20 bees each. Bees were narcotized with carbon dioxide and each subgroup was placed in a separate wax-coated paper cup (177 cm^3^; Solo S306, Highland Park, IL) covered with cotton cheesecloth and secured with two rubber bands. A maximum sublethal pre-treatment of an acaricide, fungicide, enzyme inhibitor, or solvent control, was then applied to bees in all subgroups. Model enzyme inhibitor pre-treatments were applied to bees at the following doses: 100 µg DEM, 10 µg DEF or 10 µg PBO [35]. Sublethal doses used as pre-treatment for acaricides were determined in preliminary bioassays or previous studies [22] and corresponded to doses less than or near the LD_10_ values determined in the current experiment. Acaricide pre-treatments applied to each bee were: 1 µg tau-fluvalinate, 3 µg coumaphos, 1 µg fenpyroximate, 1 µg amitraz, 10 µg thymol and 100 µg oxalic acid. Sublethal fungicide pre-treatment doses for each bee were: 10 µg chlorothalonil, 10 µg prochloraz, 10 µg pyraclostrobin, 20 µg boscalid, and 10 µg pyraclostrobin together with 20 µg boscalid to replicate the ratio of active ingredients in Pristine. All doses were delivered topically in 1 µl of acetone and applied to the thoracic notum using a 50 µl syringe fitted in a repeating dispenser (Hamilton PB-600, Reno, NV). A control pre-treatment consisting of 1 µl of pure acetone was also included.

Bioassays testing the potential for interactive effects between tau-fluvalinate and three different doses of the SBI fungicides, prochloraz, fenbuconazole, metconazole, myclobutanil, prochloraz and propiconazole, plus PBO, were performed in 2010. To standardize for the different molecular weights of PBO and the SBI fungicides bees were pre-treated at eqimolar dose levels: 0.1, 1 or 10 nanomoles fungicide per bee (corresponding to approximately 0.03, 0.3 and 3 µg per bee). Acetone was also applied as a pre-treatment control.

Bees receiving formulated antimicrobial drug pre-treatments were fed Duramycin-10 (40% oxytetracycline, Durvet, Blue Springs, MO, USA), Tylan (100% tylosin, Elanco, Greenfield, IN, USA) and Fumagilin-B (2.1% fumagillin, Medivet, Mandeville, LA, USA), dissolved in 50% sucrose water and fed to bees at a concentration of 1 mg/ml oxytetracycline active ingredient (a.i.), 1 mg/ml tylosin (a.i.) and 0.5 mg/ml fumagillin (a.i.). Drug feeding began 24 h prior to acaricide treatment and continued until mortality was scored. The sublethal concentrations used are similar to label recommendations for application of these drugs to whole colonies. The drugs in 50% sucrose water, and a sucrose water control, were fed to large groups of bees for the first 24 h of exposure through 20 ml glass scintillation vials covered with cotton cheesecloth, which were weighed before and after feeding to determine drug consumption. After acaricide treatment drugs in 50% sucrose water were provided in punctured 1.5 ml microcentrifuge tubes.

### Acaricide Dose-response Determination

Groups of pre-treated bees were allowed to recover for 1 h to minimize mortality due to extended carbon dioxide anaesthetization. Each subgroup of approximately 20 bees, of the 8–13 dose-groups in the bioassay, was then anaesthetized again and treated topically with either an acetone solvent control or one of a range of 7–12 acaricide doses, including doses eliciting 0% and 100% mortality, and at least three doses causing intermediate mortality. After treatment sucrose water was provided to groups of treated bees through a punctured 1.5 ml microcentrifuge tube following dosing, and bees were returned to the 34°C incubator. Mortality was scored at 24 h after treatment with all acaricides except fenpyroximate. Preliminary experiments showed that mortality was similar at both 24 and 48 h following treatment for acaricides except fenpyroximate. Immobile bees were scored as dead. Treatment series with greater than 5% mortality in the solvent control group were removed from analyses. At least three replicate treatment series were performed for each combination of pre-treatment and acaricide. Separately diluted acaricide dose series and bees taken from different colonies were used for each replicate treatment.

### Statistical Analyses

**Figure 3:**
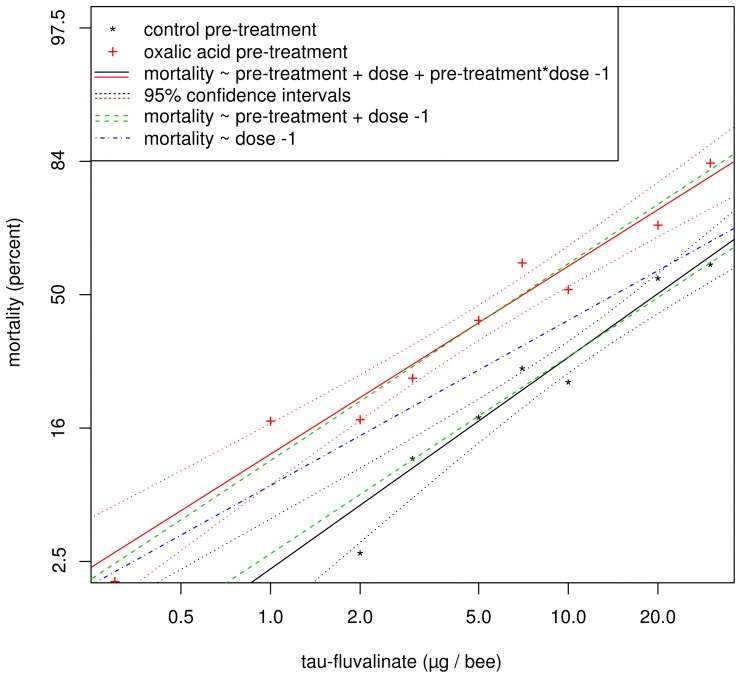
Plot of log-transformed dose and probit-transformed honey bee mortality data for tau-fluvalinate toxicity after oxalic acid or control pre-treatments. Symbols represent raw mortality and solid lines indicate lines fitted using the full model. Dotted lines represent 95% confidence intervals for each line fit with the full model. Dashed green lines were fitted using the same slope for both pre-treatments. The dashed blue line was fitted with combined data from both pre-treatments. Likelihood ratio tests comparing the full model and the reduced models were used to determine pre-treatment effects and pre-treatment * dose effects.

Lines were fitted to dose-mortality data on a log-probit scale for each pretreatment-acaricide combination using ‘glm’ in the R statistical package [36]. From these lines the lethal dose 50% (LD_50_) values and accompanying 95% confidence intervals were calculated using Fieller’s method, with correction for heterogeneity where appropriate [37]. Interactive effects between acaricides and the various pre-treatments were determined with pairwise tests comparing the dose-response lines for bees receiving pre-treatments with bees receiving a control pre-treatment using a test analogous to ANCOVA [26]. The full model, which includes the dose of acaricide as a covariate, pre-treatment as a categorical factor and the interaction between acaricide dose and pre-treatment, was compared with two simplified models (Figure 3). A process of model simplification was undertaken in which the explanatory power of model terms was assessed by reference to the likelihood ratio [38]. The first simplified model lacks the interaction term and tests the interaction between pre-treatment and acaricide dose, essentially testing for differences in the slope of the two dose-response lines (also known as a test of the “hypothesis of parallelism” [26]). A significant change in slope, as determined by a significant “dose by pre-treatment” interaction, may indicate competitive inhibition between the pre-treatment and acaricide [26]. The second simplified model lacks the pre-treatment factor entirely and was used to test the effect of pre-treatment on acaricide toxicity (also known as the “hypothesis of equality” [26]). A significant pre-treatment effect is evidence of an agonistic or antagonistic interaction between the two treatments.

Statistical significance for the model comparisons was determined by comparing likelihood ratios, corrected for heterogeneity, against an F-distribution [26], [38]. P-values for the 73 pairwise comparisons were corrected using the Holm-Bonferroni procedure (Figures 1 and 2). The 15 pairwise comparisons between tau-fluvalinate and SBI fungicides were separately corrected for multiple comparisons. A sample R script showing the analysis is available (Methods S1).

## Results

The relative toxicity of topical application of the five acaricides to bees receiving the control pre-treatment, from most to least toxic, was amitraz>fenpyroximate>tau-fluvalinate>coumaphos>thymol in 2009 (Figure 1 and Table S1). In 2010, the pattern of toxicity was similar, but fenpyroximate was found to be the most toxic acaricide and amitraz the second most toxic (Figure 2 and Table S2). Tau-fluvalinate, using identical methods, was more toxic to bees in 2010 than in 2009 (based on non-overlap of 95% confidence intervals) (Figures 1 and 2). Fenpyroximate also appeared to be more toxic to bees in 2010, though bees in 2010 were not subjected to acetone pretreatment.

The different acaricides varied greatly in their propensity for interaction with other compounds. Tau-fluvalinate interacted with most other compounds tested, including all 5 acaricides, 8 of 9 fungicides or a fungicide combination, and 2 of 3 antimicrobial compounds. Fewer interactions were observed with coumaphos, fenpyroximate and thymol. Coumaphos interacted with 3 of 5 acaricides, 1 of 4 fungicides and none of the 3 antimicrobials. Fenpyroximate interacted with 3 of 5 acaricides, 2 of 5 fungicides and 2 of 3 antimicrobials. Thymol interacted with 3 of 5 acaricides, 2 of 5 fungicides and none of the 3 antimicrobials. Amitraz did not interact with any fungicides or antimicrobials, but did demonstrate an antagonistic interaction with a single acaricide, oxalic acid.

**Figure 4:**
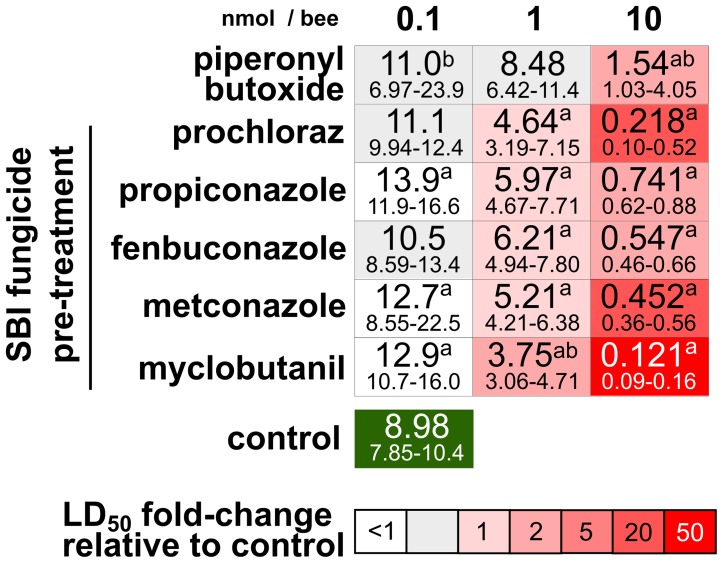
Median lethal doses (LD_50_) for tau-fluvalinate in honey bees pre-treated with piperonyl butoxide (PBO) or a sterol biosynthesis inhibiting (SBI) fungicide at three dose levels. Significant differences compared to the control treatment are indicated with a superscript letter a = significant pre-treatment effect, b = significant pre-treatment*acaricide dose effect (Table S3).

Approximately half of the acaricide-acaricide (15 of 25) and acaricide-fungicide (13 of 28) combinations tested showed evidence of interactions, nearly all of which were agonistic and resulted in increased acaricide toxicity (Figures 1 and 4). Four interactions were detected among the 15 antimicrobial-acaricide combinations tested, two of which were antagonistic interactions with fenpyroximate (Figure 2). Bees fed oxytetracycline demonstrated no change in tau-fluvalinate toxicity at the LD_50_ level, but a significant increase in the slope of the fitted dose-response line was observed. Bees fed antimicrobial drugs as a pre-treatment consumed 5.44±0.67 µl sugar water/bee/day, regardless of drug content (ANOVA, p>0.05, N = 27).

Piperonyl butoxide, a model P450 inhibitor, and DEF, a model carboxylesterase inhibitor, both increased the toxicity of fenpyroximate, but not amitraz or thymol (Figure 1).

There was no single pre-treatment that interacted with all acaricides, although oxalic acid pre-treatment caused an interactive effect with all acaricides except coumaphos. Three compounds did not interact with any acaricide tested: the antimicrobial drug tylosin, the enzyme inhibitor DEM, and the fungicide boscalid.

The most potent agonistic interaction observed in the first year of the study was between prochloraz and tau-fluvalinate (Figure 1). To determine if similar interactions occur between tau-fluvalinate and other more widely used SBI fungicides, four additional fungicides in this class (propiconazole, fenbuconazole, metconazole, and myclobutanil) were tested in combination with tau-fluvalinate (Figure 4 and Table S3) in the second year of the study. Antagonistic interactions were observed between tau-fluvalinate and 3 of 5 SBI fungicides when pre-treated with 0.1 nmol per bee. Tau-fluvalinate became more toxic (1.4 to 2.3-fold) when bees were pre-treated with any of the 5 fungicides or PBO at the 1 nmol dose, and much more toxic (12 to 74-fold) at the 10 nmol dose. An interaction between tau-fluvalinate and PBO (5.8-fold) was only observed at the 10 nmol dose level.

## Discussion

For each major group of toxicants applied in combination with acaricides – model enzyme inhibitors, acaricides, fungicides, and antimicrobials – we first consider the physiological implications of our results, especially as they relate to the involvement of detoxification enzyme systems, as well as non-transitivity that was observed in some of the interactions. Finally, we address the environmental relevance of our findings for assessing risks of the toxicants to bee health.

### Model Enzyme Inhibitor Interactions

The agonistic interactions observed between PBO and tau-fluvalinate [35], coumaphos [22] and fenpyroximate imply that P450 enzymes play a role in detoxifying these acaricides in honey bees. Heterologous expression of three honey bee P450 enzymes demonstrated that P450s are capable of metabolizing both tau-fluvalinate and coumaphos *in vitro* [39]. Agonistic interactions with DEF suggest that tau-fluvalinate, coumaphos and fenpyroximate are also detoxified through carboxylesterase activity. No interactions were observed between DEM and the acaricides. Therefore, either glutathione-S-transferase enzyme activity is not important for the detoxication of acaricides or DEM is not an effective inhibitor of the relevant enzymes in honey bees.

Thymol and amitraz did not interact with any of the inhibitors, which indicates that detoxication is not important for bees’ tolerance of these compounds. However, despite their unchanged toxicity in the presence of enzyme inhibitors, both thymol and amitraz have the potential to interact with detoxicative enzymes [40], [41] and may interfere with detoxication of other xenobiotics.

### Acaricide-acaricide Interactions

The cluster of interactions observed between tau-fluvalinate, coumaphos and fenpyroximate, all of which appear to be detoxified by P450s and carboxylesterases, suggest that interactions between these compounds may be occurring because these acaricides interact with detoxicative enzymes [22], [39]. However, not every combination of these acaricides caused an interaction. Since acaricides were used both as treatments and as pre-treatments, we were able to investigate the temporal transitivity of the interactions. A combination is ‘transitive’ if the same effect occurs irrespective of the order of exposure and ‘non-transitive’ if an interaction is only observed when one of the pair of compounds is applied first. A non-transitive interaction was observed between tau-fluvalinate and fenpyroximate. While fenpyroximate pre-treatment increased the toxicity of tau-fluvalinate 8-fold, the opposite was not true. Tau-fluvalinate pre-treatment did not significantly change the toxicity of fenpyroximate. The non-transitivity exhibited between fenpyroximate and tau-fluvalinate may indicate that fenpyroximate can competitively inhibit the specific P450 isozymes involved in tau-fluvalinate detoxication [39] while tau-fluvalinate does not interact with the specific P450s that allow bees to tolerate fenpyroximate exposure.

The toxicity of thymol increased following pre-treatment with either tau-fluvalinate or coumaphos. Since it is not known how bees tolerate thymol, it is difficult to speculate about the basis for these interactions. Coumaphos toxicity was unchanged by thymol, but tau-fluvalinate toxicity was transitively increased when thymol was applied as a pre-treatment. Monoterpenoids, like thymol, have been shown to inhibit P450 activity in human liver microsomes [40], so this agonistic interaction may be a result of thymol inhibition of the P450s on which tolerance of tau-fluvalinate depends.

Amitraz participated in three non-transitive acaricide-acaricide interactions. The toxicity of amitraz was unchanged when bees were pre-treated with tau-fluvalinate. However, amitraz pre-treatment increased the toxicity of tau-fluvalinate 5-fold. Interactions between formamidines and pyrethroids are known in other insects and may be due to synergism at the target site through cooperative binding [42], or through inhibition of pyrethroid detoxication, through inhibition of P450s or carboxylesterases [41].

Amitraz is bioactivated through a hydrolysis reaction to DMPF which is an octopamine receptor agonist [29], [43]. Bees may tolerate amitraz as an acaricide because they are poor at amitraz bioactivation, possibly due to the paucity of genes that encode P450s and carboxylesterases in the honey bee genome [44]. Despite the apparent lack of amitraz bioactivation in bees, this compound may have some affinity for the P450 enzymes that are important for tau-fluvalinate and fenpyroximate detoxication.

In one of the few antagonistic interactions observed, oxalic acid pre-treatment reduced the toxicity of amitraz nearly four-fold. However, this interaction may be an artifact of the methods used. Amitraz was applied to the same location on the thoracic notum as the oxalic acid pre-treatment, which may have caused degradation of amitraz, which is known to undergo hydrolytic breakdown under acidic conditions [45], while tau-fluvalinate and coumaphos do not [46]. All other acaricides showed some agonistic effects with oxalic acid, possibly due to increased cuticular penetration caused by the abrasive effect of oxalic acid crystals on the epicuticle or because of the elevated production of reactive oxygen species initiated by oxalic acid [47], [48]. Future studies may seek to avoid the effects of local site interactions by using different application sites or different routes of administration.

### Environmental Relevance of the Observed Acaricide-acaricide Interactions

High sublethal doses were deliberately chosen as pre-treatments to make the magnitude of any interactive effect between the pre-treatment and acaricide as large as possible. However, to determine the hazard posed by acaricide interactions in bee hives, the actual exposure bees receive when treated with formulated acaricides must be considered. Margins of safety for each acaricide formulation can be estimated by dividing the dose estimated to cause 10% mortality (LD_10_) by the estimated daily dose. The LD_10_ can be determined from the fitted dose response lines (Table S1). The daily dose can be estimated by first determining the amount of active ingredient present in each acaricide formulation, correcting this value by the amount of active ingredient available for uptake by the bees (e.g. 10% of the total acaricide content in strip formulations is taken up by bees [49], [50]) and dividing this quantity by the number of days the acaricide formulation is present in the hive. The estimated daily dose calculated in this way can be validated using published pesticide residue data [3], [51]. Based on these calculations, coumaphos and tau-fluvalinate formulations carry the largest margins of safety for honey bees (43- and 29-fold, respectively), while fenpyroximate, amitraz, thymol and oxalic acid formulations have narrower margins of safety (2.0 to 7.8-fold). A consequence of the great differences in margins of safety between acaricides is that the potent interactions observed in this study with tau-fluvalinate and coumaphos may be less harmful in the real world than apparently milder interactions observed with fenpyroximate and thymol.

Beeswax is the ultimate sink for the lipophilic pesticides in the beehive and beeswax may contain up to 94 ppm coumaphos and 204 ppm tau-fluvalinate [3], [49]. Both coumaphos and tau-fluvalinate are quite stable in the wax component of the hive where they can persist for years and even increase in concentration when acaricide treatments are repeated [50], [52], [53]. Bees are also exposed to the bioactivated breakdown product of amitraz, DPMF, at concentrations as high as 43 ppm in wax [3]. Thymol has been detected in wax at concentrations as high as 4753 ppm [12]. Coumaphos and tau-fluvalinate survive the wax recycling process and are present in newly manufactured wax foundation [3], [12]. Acaricide contamination is pervasive in managed colonies, but it is unknown what fraction of these compounds can leach out of wax and into adult bees and developing brood. The burden of acaricides and other pesticides in old wax brood comb is sufficient to reduce brood survival [54], delay the development time of larvae and reduce the lifespan of adult workers [55], and increase the susceptibility of adults to *Nosema* infection [56]. Combinatorial effects between acaricides and other pesticides present in the wax [3] may be contributing to the observed negative effects of old comb.

### Fungicide-acaricide Interactions

In 2009 experiments the SBI fungicide prochloraz produced an almost 2000-fold increase in the toxicity of tau-fluvalinate. Tau-fluvalinate, coumaphos and fenpyroximate – all acaricides for which P450-mediated detoxication was implicated through synergistic interactions with PBO – also showed a synergistic interaction with prochloraz. These results confirm previous reports of interactions between pyrethroid insecticides and prochloraz through P450s [20], and extend those findings to show that this fungicide can affect the metabolism of acaricides as well as field-applied pesticides.

Because of the potent interaction observed between prochloraz and tau-fluvalinate, additional P450-interacting SBI fungicides were tested that are extensively used in a variety of cropping systems. The SBI family of fungicides are known to be present in bee-collected pollen, wax and in bees themselves [2], [3], [57]. Five SBI fungicides demonstrated synergism with the pyrethroids alpha-cypermethrin and lambda-cyhalothrin in bees [23]. We found that a similar suite of SBI fungicides could interact synergistically with the pyrethroid tau-fluvalinate, likely through inhibition of P450s, when administered at 1 or 10 nmol of fungicide per bee. Unexpectedly, an antagonistic interaction was observed at the lowest fungicide doses (0.1 nmol or approximately 0.03 µg per bee). This antagonism suggests that, in addition to inhibiting bee P450 enzymes, the SBI fungicides may also induce P450 gene expression at low levels of exposure, including the genes encoding P450 isozymes that are involved in tau-fluvalinate detoxication [39]. Low doses of P450 inhibitors have been found to induce P450 gene expression in other insects [58] and SBI fungicides can induce P450 enzyme activity in mammals [59]. Quercetin, a pollen flavonol that is known to interact with bee P450s [60], caused a similar reduction in tau-fluvalinate toxicity in bees [61].

Pre-treatment of bees with the fungicide chlorothalonil increased the toxicity of both thymol and tau-fluvalinate. Chlorothalonil is a multi-site action fungicide in the chloronitrile family and is metabolized through P450 activity to 4-hydroxy-2,5,6-trichloroisophthalonitrile. This metabolite is both more toxic and more likely to cause oxidative stress than the parent compound [62], [63]. Competitive P450 inhibition of tau-fluvalinate detoxication may account for this observed interaction as well. Chlorothalonil has previously been shown to have an interactive effect with alpha-cypermethrin and lambda-cyhalothrin, two pyrethroids, in bees [23].

Pyraclostrobin and boscalid applied together in a blend like Pristine moderately increased the toxicity of tau-fluvalinate. When applied individually, however, only pyraclostrobin had an effect. Both boscalid and pyraclostrobin kill fungi through the inhibition of respiration by blocking mitochondrial electron transfer at complexes II and III, respectively [64], [65]. Pyraclostrobin also moderately increased the toxicity of fenpyroximate, which is itself an inhibitor of mitochondrial respiration at complex I [66]. Fenpyroximate, pyraclostrobin and boscalid in combination have been hypothesized to harm bees by starving them of energy through inhibition of oxidative phosphorylation. Alternatively, the interaction observed between fenpyroximate and pyraclostrobin may be the product of increased oxidative stress caused by their interference with mitochondrial electron transport [67]. Increased mortality related to oxidative stress may also explain the interaction observed between tau-fluvalinate and pyraclostrobin, as pyrethroids can also cause increased production of reactive oxygen species [68]. Other respiration-inhibiting fungicides including iprodione and vinclozlin [32] may also be of concern as these compounds have been found at high concentration in chemical analyses of bees and bee products [3].

### Environmental Relevance of Fungicide-acaricide Interactions

Consumption of contaminated pollen is the most likely route of exposure to fungicides [57]. Assuming a colony consumes an average of 20 kg of pollen per year to rear 150,000 bees or about 130 mg pollen consumed for each bee [69] the typical fungicide exposure a bee receives through pollen can be estimated. Assuming that most pollen is eaten by nurse bees during the first 10 days of adulthood [69], a nurse bee could be exposed to as much as 1.3 µg chlorothalonil per day by consuming 13 mg of pollen containing 99 ppm chlorothalonil [3]. While this dose is approximately one-eighth the dose of chlorothalonil applied in this study, we speculate that it may be sufficient to interact with tau-fluvalinate and thymol. The thymol-chlorothalonil interaction is of particular concern given the relatively low estimated margin of safety afforded by thymol formulations (Table 1).

**Table 1:** Estimated margins of safety for six acaricides based on estimated daily dosages per bee and the estimated LD_10_.

formulatedproduct	active ingredient	g	treatment duration(days)	predicted dailyexposure(µg/bee)	reported meanexposure(µg/bee)	max.(µg/bee)	LD_10_(µg/bee)	estimated marginof safety
Apistan	tau-fluvalinate	1.4	56	0.13	0.04^a^	0.70^a^	3.08	24
Checkmite+	coumaphos	2.8	45	0.31	0.09^a^	3.20^b^	12.1	39
Hivastan	fenpyroximate	0.7	42	0.40	−	−	2.19	5.5
Apivar	amitraz	1.0	42	0.12	0.15^a^	9.04^a^	0.92	7.8
ApilifeVar	thymol	49	32	7.6	−	−	15.4	2.0
Apiguard	thymol	25	28	4.5	−	−	15.4	3.4
	oxalic acid	1.8	1	44	−	−	176.7c	4.0

For reported concentrations of acaricides in bees: a = [3], and b = [51]. See the text for methods used to estimate daily exposure. Margins of safety are calculated by dividing the LD_10_ dose (Table S1 and c = [76]), by the predicted daily dose.

Pollen has been found to contain as much as 1 ppm boscalid and 0.26 ppm pyraclostrobin [3]. Nurse bees consuming this pollen may take in as much as 0.015 µg and 0.003 µg per day of boscalid and pyraclostrobin, respectively, which is three orders of magnitude less than the doses of boscalid and pyraclostrobin that demonstrated agonistic interaction with acaricides in this study. Given the mild interactions observed between these fungicides and the acaricides, and the relatively low level of these fungicides found in pollen [3], it seems unlikely to us that adult bees would suffer acute mortality from Pristine™-acaricide combinations under field conditions.

The SBI fungicides are also known to enter the hive through contaminated pollen [57]. Mullin *et al*. [3] detected the SBI fungicide myclobutanil at levels as high as approximately 1 ppm in pollen. At this concentration a nurse bee would be expected to consume 0.013 µg of myclobutanil per day, which corresponds to about half the lowest dose of this fungicide administered. A single-dose exposure to myclobutanil at this level could counter intuitively protect the bee, to some extent, from tau-fluvalinate toxicity, possibly through induction of detoxicative enzymes. However, chronic dietary exposure at this level could lead to potent synergistic interactions, as we observed at higher fungicide doses, depending at the rate at which myclobutanil is metabolized or excreted.

### Antimicrobial-acaricide Interactions

Simultaneous exposure to tau-fluvalinate and oxytetracycline, which blocks multi-drug resistance transporters, has been shown in previous work to increase tau-fluvalinate toxicity [70]. In our testing, pretreating bees with oxytetracycline had no significant effect on the LD_50_ of tau-fluvalinate; however, the “pre-treatment by acaricide dose” effect was significant, indicating that the slope is significantly steeper for bees receiving oxytetracycline treatment (Table S1). This may be evidence of oxytetracycline effects on the drug transporter enzymes [70]. While amitraz and oxytetracycline have been shown to induce programmed cell death in the honey bee midgut [71], this combination had no effect on acute mortality.

An antagonistic interaction was observed between fenpyroximate and oxytetracycline. Oxytetracycline has been reported to have antioxidant properties [72], which may ameliorate the effects of fenpyroximate, a pesticide known to cause oxidative-stress in mammals [67].

### Between-year Variability

Honey bees treated in 2009 were more tolerant of tau-fluvalinate than bees in both previous studies [22] and bees treated in 2010. A study conducted in 2008 using an identical protocol determined an LD_50_ for tau-fluvalinate of 6.75 µg per bee [22]. The LD_50_ for tau-fluvalinate in 2009 was determined to be 19.8 µg per bee, which is substantially higher than 9.2 µg and 9.0 µg per bee determined in 2010 for oral and topical control pre-treatments, respectively. This substantial difference in susceptibility to tau-fluvalinate and other acaricides between years occurred despite the fact that bees were kept in the same apiary, in the same woodenware, and were collected for bioassays over the same months of the year and, for the most part, were subjected to bioassays by the same workers using the same equipment and technical grade tau-fluvalinate purchased from the same source. The only obvious difference between years was the genetic stock as queens in all colonies were replaced at the beginning of the season in 2010. Elzen *et al*. [73], noted that European honey bees (*A. mellifera ligustica*) were significantly more tolerant of the pyrethroid cyfluthrin than African honey bees (*A. mellifera scutellata*) and suggested that this difference may be the product of inadvertent selection for pyrethroid tolerance resulting from the widespread use of tau-fluvalinate as an acaricide in managed European honey bee colonies. In the same manner, the observed difference in tau-fluvalinate toxicity between populations of European honey bees may be due to inadvertent selection for tolerance through queen breeders’ choice of acaricides.

### Conclusions

Detoxication by P450s appears to be the basis for the tolerance bees show toward tau-fluvalinate, coumaphos and fenpyroximate, but not amitraz or thymol. Any SBI fungicide that inhibits P450-mediated detoxification has the potential to interact with tau-fluvalinate, and likely coumaphos and fenpyroximate as well. Given the large number of pesticides to which bees are potentially exposed, and the practical impossibility of testing every possible combination, examining pesticide interaction with P450s holds promise as a method to simplify pesticide interaction testing in a rational way. Testing for P450 interactions could serve as the first “tool” in a lab-based “toolbox” testing for potential pesticide interactions in bees.

While these laboratory bioassays point to potential problems associated with the various acaricide treatments, any management recommendations must be based on additional information gained from field experiments using whole colonies. Lethal-dose bioassays, by definition, require the use of doses that are high enough to cause acute mortality in bees – doses that are often much higher than bees are likely to encounter under field conditions. However, documentation of these interactions provides a foundation for future experiments using field-relevant doses and helps to focus the limited resources available for field experiments on those pesticide combinations with the greatest potential to cause harm. The routes of exposure to acaricides, fungicides and antimicrobials in beehives may be different from the topical and oral applications used in these bioassays. The actual exposure bees receive to formulated acaricides, sequestered acaricides in beeswax, and fungicide applications in agriculture need to be quantified to accurately assess the risk posed by interactions. Additionally, bees may experience sublethal effects that are, by definition, not quantifiable in lethal dose bioassays, but may have a substantial effect on colony health [74], [75]. Until more is known about the potential for interaction between acaricides, fungicides and antimicrobials in the real world it would be prudent for beekeepers to avoid concurrent use of acaricides that are detoxified by P450s – tau-fluvalinate, coumaphos, and fenpyroximate – especially in settings where honey bees may be simultaneously exposed to the P450-inhibiting SBI fungicides.

## Supporting Information

Table S1 **Dose-response line parameters and pairwise comparisons for topical application of five acaricides following sublethal topical pre-treatment with another acaricide, fungicide or detoxicative enzyme inhibitor.**
(DOCX)

Table S2 **Dose-response line parameters and pairwise comparisons for topical application of five acaricides following 24 h oral treatment with antimicrobial drugs.**
(DOCX)

Table S3 **Dose-response line parameters and pairwise comparisons for tau-fluvalinate following treatment with sterol biosynthesis inhibiting fungicides.**
(DOCX)

Methods S1 **Sample R script for lethal dose determination and pairwise comparison of two dose-response curves.**
(DOC)

## References

[1] Bogdanov S (2006) Contaminants of bee products. Apidologie 37: 1–18 doi:10.1051/apido:2005043.

[2] Chauzat MP, Faucon JP (2007) Pesticide residues in beeswax samples collected from honey bee colonies (*Apis mellifera* L.) in France. Pest Manag Sci 63: 1100–1106 doi:10.1002/ps.1451. 17879980 10.1002/ps.1451

[3] Mullin CA, Frazier M, Frazier JL, Ashcraft S, Simonds R, et al (2010) High levels of miticides and agrochemicals in North American apiaries: implications for honey bee health. PLoS ONE 5: e9754 doi:10.1371/journal.pone.0009754. 20333298 10.1371/journal.pone.0009754 PMC2841636

[4] Rosenkranz P, Aumeier P, Ziegelmann B (2010) Biology and control of *Varroa destructor* . J Invertebr Pathol 103: S96–S119 doi:10.1016/j.jip.2009.07.016. 19909970 10.1016/j.jip.2009.07.016

[5] Le Conte Y, Ellis MD, Ritter W (2010) Varroa mites and honey bee health: can Varroa explain part of the colony losses? Apidologie 41: 353–363 doi:10.1051/apido/2010017.

[6] Genersch E, von der Ohe W, Kaatz H, Schroeder A, Otten C, et al (2010) The German bee monitoring project: a long term study to understand periodically high winter losses of honey bee colonies. Apidologie 41: 332–352 doi:10.1051/apido/2010014.

[7] Boecking O, Genersch E (2008) Varroosis - the ongoing crisis in bee keeping. J Verbrauch Lebensm 3: 221–228 doi:10.1007/s00003-008-0331-y.

[8] vanEngelsdorp D, Evans JD, Saegerman C, Mullin C, Haubruge E, et al (2009) Colony collapse disorder: a descriptive study. PLoS ONE 4: e6481 doi:10.1371/journal.pone.0006481. 19649264 10.1371/journal.pone.0006481 PMC2715894

[9] Lodesani M, Colombo M, Spreafico M (1995) Ineffectiveness of Apistan® treatment against the mite Varroa jacobsoni Oud in several districts of Lombardy (Italy). Apidologie 26: 67–72 doi:10.1051/apido:19950109.

[10] Elzen PJ, Westervelt D (2002) Detection of coumaphos resistance in *Varroa destructor* in Florida. Am Bee J 142: 291–292.

[11] Bogdanov S (2004) Beeswax: quality issues today. Bee World 85: 46–50.

[12] Bogdanov S, Imdorf A, Kilchenmann V (1998) Residues in wax and honey after Apilife VAR® treatment. Apidologie 29: 513–524 doi:10.1051/apido:19980604.

[13] Martel AC, Zeggane S, Aurières C, Drajnudel P, Faucon JP, et al (2007) Acaricide residues in honey and wax after treatment of honey bee colonies with Apivar® or Asuntol® 50. Apidologie 38: 534–544 doi:10.1051/apido:2007038.

[14] Rademacher E, Harz M (2006) Oxalic acid for the control of varroosis in honey bee colonies – a review. Apidologie 37: 98–120 doi:10.1051/apido:2005063.

[15] Adamczyk S, Lázaro R, Pérez-Arquillué C, Conchello P, Herrera A (2005) Evaluation of residues of essential oil components in honey after different anti-Varroa treatments. J Agric Food Chem 53: 10085–10090 doi:10.1021/jf051813f. 16366699 10.1021/jf051813f

[16] Mussen EC (2008) Fungicides toxic to bees? From the UC Apiaries 17: 1–3.

[17] Atkins EL (1992) Injury to honey bee by poisoning. In: Graham JE, editor. The Hive and the Honey Bee. Hamilton, IL: Dadant and Sons. 1153–1208.

[18] Mussen EC, Lopez JE, Peng CYS (2004) Effects of selected fungicides on growth and development of larval honey bees, *Apis mellifera* L. (Hymenoptera: Apidae). Environ Entomol 33: 1151–1154 doi:10.1603/0046-225X-33.5.1151.

[19] vanEngelsdorp D, Evans JD, Donovall L, Mullin C, Frazier M, et al (2009) “Entombed pollen”: a new condition in honey bee colonies associated with increased risk of colony mortality. J Invertebr Pathol 101: 147–149 doi:10.1016/j.jip.2009.03.008. 19361513 10.1016/j.jip.2009.03.008

[20] Pilling ED, Bromley-Challenor KAC, Walker CH, Jepson PC (1995) Mechanism of synergism between the pyrethroid insecticide λ-Cyhalothrin and the imidazole fungicide prochloraz, in the honeybee (*Apis mellifera* L.). Pestic Biochem Physiol 51: 1–11 doi:10.1006/pest.1995.1001.

[21] Vandame R, Belzunces LP (1998) Joint actions of deltamethrin and azole fungicides on honey bee thermoregulation. Neurosci Lett 251: 57–60 doi:10.1016/S0304-3940(98)00494-7. 9714464 10.1016/s0304-3940(98)00494-7

[22] Johnson RM, Pollock HS, Berenbaum MR (2009) Synergistic interactions between in-hive miticides in *Apis mellifera* . J Econ Entomol 102: 474–479 doi:10.1603/029.102.0202. 19449624 10.1603/029.102.0202

[23] Thompson H, Wilkins S (2003) Assessment of the synergy and repellency of pyrethroid/fungicide mixtures. Bull Insectology 56: 131–134.

[24] Colin ME, Belzunces LC (1992) Evidence of synergy between prochloraz and deltamethrin in *Apis mellifera* L: a convenient biological approach. Pestic Sci 36: 115–119 doi:10.1002/ps.2780360206.

[25] Bliss CI (1939) The toxicity of poisons applied jointly. Ann Appl Biol 26: 585–615 doi:10.1111/j.1744-7348.1939.tb06990.x.

[26] Robertson JL, Savin NE, Preisler HK, Russell RM (2007) Bioassays with arthropods. Boca Raton: CRC Press. 224 p.

[27] Garcia D, Bujons J, Vale C, Suñol C (2006) Allosteric positive interaction of thymol with the GABAA receptor in primary cultures of mouse cortical neurons. Neuropharmacology 50: 25–35 doi:10.1016/j.neuropharm.2005.07.009. 16185724 10.1016/j.neuropharm.2005.07.009

[28] Johnson RM, Ellis MD, Mullin CA, Frazier M (2010) Pesticides and honey bee toxicity – USA. Apidologie 41: 312–331 doi:10.1051/apido/2010018.

[29] Yu SJ (2008) The toxicology and biochemistry of insecticides. Baton Roca: CRC Press. 296 p.

[30] Sanders W, Sanders C (1979) Toxicity of antibacterial agents: mechanism of action on mammalian cells. Annu Rev Pharmacol Toxicol 19: 53–83 doi:10.1146/annurev.pa.19.040179.000413. 222202 10.1146/annurev.pa.19.040179.000413

[31] Zhang Y, Yeh J, Mara A, Ju R, Hines J, et al (2006) A chemical and genetic approach to the mode of action of fumagillin. Chem Biol 13: 1001–1009 doi:10.1016/j.chembiol.2006.07.010. 16984890 10.1016/j.chembiol.2006.07.010 PMC2583369

[32] Fungicide Resistance Action Committee (2012) FRAC Code List 2012. Available: http://www.frac.info/frac/publication/anhang/FRAC-Code-List2011-final.pdf Accessed: 24 August 2012.

[33] Insecticide Resistance Action Committee (2012) In:Sparks T, Salgado V, editors. MoA Classification Feb 2012. Available: http://www.irac-online.org/content/uploads/MoA-classification.pdf Accessed: 11 December 2012.

[34] Tenson T, Lovmar M, Ehrenberg M (2003) The mechanism of action of macrolides, lincosamides and streptogramin B reveals the nascent peptide exit path in the ribosome. J Mol Biol 330: 1005–1014 doi:10.1016/S0022-2836(03)00662-4. 12860123 10.1016/s0022-2836(03)00662-4

[35] Johnson RM, Wen Z, Schuler MA, Berenbaum MR (2006) Mediation of pyrethroid insecticide toxicity to honey bees (Hymenoptera: Apidae) by cytochrome P450 monooxygenases. J Econ Entomol 99: 1046–1050 doi:10.1603/0022-0493-99.4.1046. 16937654 10.1603/0022-0493-99.4.1046

[36] R Development Core Team (2012) R: A language and environment for statistical computing. Vienna, Austria: R Foundation for Statistical Computing.

[37] Finney DJ (1971) Probit analysis. Cambridge: Cambridge University Press. 350 p.

[38] Savin NE, Robertson JL, Russell RM (1977) A critical evaluation of bioassay in insecticide research: likelihood ratio tests of dose-mortality regression. Bull ESA 23: 257–266.

[39] Mao W, Schuler M, Berenbaum M (2011) CYP9Q-mediated detoxification of acaricides in the honey bee (*Apis mellifera*). Proc Natl Acad Sci U S A doi:10.1073/pnas.1109535108. 10.1073/pnas.1109535108 PMC3150950 21775671

[40] De-Oliveira AC, Fidalgo-Neto AA, Paumgartten FJ (1999) In vitro inhibition of liver monooxygenases by beta-ionone, 1,8-cineole, (−)-menthol and terpineol. Toxicology 135: 33–41 doi:10.1016/S0300-483X(99)00043-8. 10454222 10.1016/s0300-483x(99)00043-8

[41] Usmani AK, Abd-Elghafar SF, Knowles CO (1995) Amitraz effect on the pharmacokinetics of permethrin in *Helicoverpa zea* (Lepidoptera: Noctuidae). J Econ Entomol 88: 1580–1585.

[42] Liu MY, Plapp FW Jr (1992) Mechanism of formamidine synergism of pyrethroids. Pestic Biochem Physiol 43: 134–140 doi:10.1016/0048-3575(92)90027-W.

[43] Orr GL, Orr N, Cornfield L, Gole JWD, Downer RGH (1990) Interaction of formamidine pesticides with insect neural octopamine receptors: effects on radioligand binding and cyclic AMP production. Pestic Sci 30: 285–294 doi:10.1002/ps.2780300305.

[44] Claudianos C, Ranson H, Johnson RM, Biswas S, Schuler MA, et al (2006) A deficit of detoxification enzymes: pesticide sensitivity and environmental response in the honeybee. Insect Mol Biol 15: 615–636 doi:10.1111/j.1365-2583.2006.00672.x. 17069637 10.1111/j.1365-2583.2006.00672.x PMC1761136

[45] Corta E, Bakkali A, Berrueta LA, Gallo B, Vicente F (1999) Kinetics and mechanism of amitraz hydrolysis in aqueous media by HPLC and GC-MS. Talanta 48: 189–199 doi:10.1016/S0039-9140(98)00237-9. 18967458 10.1016/s0039-9140(98)00237-9

[46] Corta E, Bakkali A, Barranco A, Berrueta LA, Gallo B, et al (2000) Study of the degradation products of bromopropylate, chlordimeform, coumaphos, cymiazole, flumethrin and tau-fluvalinate in aqueous media. Talanta 52: 169–180 doi:10.1016/S0039-9140(00)00333-7. 18967974 10.1016/s0039-9140(00)00333-7

[47] Cao LC, Honeyman TW, Cooney R, Kennington L, Scheid CR, et al (2004) Mitochondrial dysfunction is a primary event in renal cell oxalate toxicity. Kidney Int 66: 1890–1900 doi:10.1111/j.1523-1755.2004.00963.x. 15496160 10.1111/j.1523-1755.2004.00963.x

[48] Meimaridou E, Jacobson J, Seddon AM, Noronha-Dutra AA, Robertson WG, et al (2005) Crystal and microparticle effects on MDCK cell superoxide production: oxalate-specific mitochondrial membrane potential changes. Free Radic Biol Med 38: 1553–1564 doi:10.1016/j.freeradbiomed.2005.02.020. 15917184 10.1016/j.freeradbiomed.2005.02.020

[49] Tremolada P, Bernardinelli I, Colombo M, Spreafico M, Vighi M (2004) Coumaphos distribution in the hive ecosystem: case study for modeling applications. Ecotoxicology 13: 589–601 doi:10.1023/B:ECTX.0000037193.28684.05. 15526863 10.1023/b:ectx.0000037193.28684.05

[50] Bogdanov S, Kilchenmann V, Imdorf A (1998) Acaricide residues in some bee products. J Apic Res 37: 57–67.

[51] Haarmann T, Spivak M, Weaver D, Weaver B, Glenn T (2002) Effects of fluvalinate and coumaphos on queen honey bees (Hymenoptera: Apidae) in two commercial queen rearing operations. J Econ Entomol 95: 28–35 doi:10.1603/0022-0493-95.1.28. 11942761 10.1603/0022-0493-95.1.28

[52] Sokol R (1996) The influence of a multimonth persistence of Fluwarol in a hive of a honey bee colony. Medycyna Weterynaryjna 52: 718–720.

[53] Wallner K (1999) Varroacides and their residues in bee products. Apidologie 30: 235–238 doi:10.1051/apido:19990212.

[54] Medici SK, Castro A, Sarlo EG, Marioli JM, Eguaras MJ (2012) The concentration effect of selected acaricides present in beeswax foundation on the survival of *Apis mellifera* colonies. J Apic Res 51: 164–168 doi:10.3896/IBRA.1.51.2.03.

[55] Wu JY, Anelli CM, Sheppard WS (2011) Sub-lethal effects of pesticide residues in brood comb on worker honey bee (*Apis mellifera*) development and longevity. PLoS ONE 6: e14720 doi:10.1371/journal.pone.0014720. 21373182 10.1371/journal.pone.0014720 PMC3044129

[56] Wu JY, Smart MD, Anelli CM, Sheppard WS (2012) Honey bees (*Apis mellifera*) reared in brood combs containing high levels of pesticide residues exhibit increased susceptibility to *Nosema* (Microsporidia) infection. J Invert Path 109: 326–329 doi:10.1016/j.jip.2012.01.005. 10.1016/j.jip.2012.01.005 22285445

[57] Kubik M, Nowacki J, Pidek A, Warakomska Z, Michalczuk L, et al (2000) Residues of captan (contact) and difenoconazole (systemic) fungicides in bee products from an apple orchard. Apidologie 31: 531–541 doi:10.1051/apido:2000144.

[58] Willoughby L, Batterham P, Daborn P (2007) Piperonyl butoxide induces the expression of cytochrome P450 and glutathione S-transferase genes in *Drosophila melanogaster* . Pest Manag Sci 63: 803–808 doi:10.1002/ps.1391. 17514638 10.1002/ps.1391

[59] Sergent T, Dupont I, Jassogne C, Ribonnet L, van der Heiden E, et al (2009) CYP1A1 induction and CYP3A4 inhibition by the fungicide imazalil in the human intestinal Caco-2 cells–comparison with other conazole pesticides Toxicol Lett. 184: 159–168 doi:10.1016/j.toxlet.2008.11.009. 10.1016/j.toxlet.2008.11.009 19070657

[60] Mao W, Rupasinghe SG, Johnson RM, Zangerl AR, Schuler MA, et al (2009) Quercetin-metabolizing CYP6AS enzymes of the pollinator *Apis mellifera* (Hymenoptera: Apidae). Comp Biochem Physiol B Biochem Mol Biol 154: 427–434 doi:10.1016/j.cbpb.2009.08.008. 19737624 10.1016/j.cbpb.2009.08.008

[61] Johnson RM, Mao W, Pollock HS, Niu G, Schuler M, et al (2012) Ecologically appropriate xenobiotics induce cytochrome P450s in *Apis mellifera* . PLoS ONE 7: e31051 doi:10.1371/journal.pone.0031051. 22319603 10.1371/journal.pone.0031051 PMC3272026

[62] Chaves A, Shea D, Danehower D (2008) Analysis of chlorothalonil and degradation products in soil and water by GC/MS and LC/MS. Chemosphere 71: 629–638 doi:10.1016/j.chemosphere.2007.11.015. 18096203 10.1016/j.chemosphere.2007.11.015

[63] Suzuki T, Nojiri H, Isono H, Ochi T (2004) Oxidative damages in isolated rat hepatocytes treated with the organochlorine fungicides captan, dichlofluanid and chlorothalonil. Toxicology 204: 97–107 doi:10.1016/j.tox.2004.06.025. 15388237 10.1016/j.tox.2004.06.025

[64] Kuhn PJ (1984) Mode of action of carboximides. British Mycological Society Symposium 9: 155–183.

[65] Anke T (1995) The antifungal strobilurins and their possible ecological role. Can J Bot 73: 940–945.

[66] Motoba K, Suzuki T, Uchida M (1992) Effect of a new acaricide, fenpyroximate, on energy metabolism and mitochondrial morphology in adult female *Tetranychus urticae* (two-spotted spider mite). Pestic Biochem Physiol 43: 37–44 doi:10.1016/0048-3575(92)90017-T.

[67] Sherer TB, Richardson JR, Testa CM, Seo BB, Panov AV, et al (2007) Mechanism of toxicity of pesticides acting at complex I: relevance to environmental etiologies of Parkinson’s disease. J Neurochem 100: 1469–1479 doi:10.1111/j.1471-4159.2006.04333.x. 17241123 10.1111/j.1471-4159.2006.04333.x PMC8669833

[68] Kale M, Rathore N, John S, Bhatnagar D (1999) Lipid peroxidative damage on pyrethroid exposure and alterations in antioxidant status in rat erythrocytes: a possible involvement of reactive oxygen species. Toxicol Lett 105: 197–205 doi:10.1016/S0378-4274(98)00399-3. 10355540 10.1016/s0378-4274(98)00399-3

[69] Seeley TD (1995) The wisdom of the hive: the social physiology of honey bee colonies. Cambridge: Harvard University Press. 318 p.

[70] Hawthorne D, Dively G (2011) Killing them with kindness? In-hive medications may inhibit xenobiotic efflux transporters and endanger honey bees. PLoS ONE 6: e26796 doi:10.1371/journal.pone.0026796. 22073195 10.1371/journal.pone.0026796 PMC3206626

[71] Gregorc GA, Bowen ID (2000) Histochemical characterization of cell death in honeybee larvae midgut after treatment with *Paenibacillus larvae*, amitraz, and oxytetracycline. Cell Biol Int 24: 319–324 doi:10.1006/cbir.1999.0490. 10805966 10.1006/cbir.1999.0490

[72] Miyachi Y, Yoshioka A, Imamura S, Niwa Y (1986) Effect of antibiotics on the generation of reactive oxygen species. J Invest Dermatol 86: 449–453 doi:10.1111/1523-1747.ep12285793. 3755739 10.1111/1523-1747.ep12285793

[73] Elzen PJ, Elzen GW, Rubink W (2003) Comparative susceptibility of European and Africanized honey bee (Hymenoptera: Apidae) ecotypes to several insecticide classes. Southwestern Entomologist 28: 255–260.

[74] Dai PL, Wang Q, Sun JH, Liu F, Wang X, et al (2010) Effects of sublethal concentrations of bifenthrin and deltamethrin on fecundity, growth, and development of the honeybee *Apis mellifera ligustica* . Environ Toxicol Chem 29: 644–649 doi:10.1002/etc.67. 20821489 10.1002/etc.67

[75] Gill RJ, Ramos-Rodriguez O, Raine NE (2012) Combined pesticide exposure severely affects individual- and colony-level traits in bees. Nature 491: 105–108 doi:10.1038/nature11585. 23086150 10.1038/nature11585 PMC3495159

[76] Aliano NP, Ellis MD, Siegfried BD (2006) Acute contact toxicity of oxalic acid to *Varroa destructor* (Acari: Varroidae) and their *Apis mellifera* (Hymenoptera: Apidae) hosts in laboratory bioassays. J Econ Entomol 99: 1579–1582 doi:10.1603/0022-0493–99.5.1579. 17066785 10.1603/0022-0493-99.5.1579

