# Supplementary material for: Acaricide, Fungicide and Drug Interactions in Honey Bees (Apis mellifera)
Source: PLoS One. 2013 Jan 29;8(1):e54092. doi: 10.1371/journal.pone.0054092 (PMC3558502; doi:10.1371/journal.pone.0054092)
Supplement: Table S1 — Dose-response line parameters and pairwise comparisons for topical application of five acaricides following sublethal topical pre-treatment with another acaricide, fungicide or detoxicative enzyme inhibitor. (DOCX) [file pone.0054092.s001.docx]

**Table S1.** Dose-response line parameters and pairwise comparisons for topical application of five acaricides following sublethal topical pre-treatment with another acaricide, fungicide or detoxicative enzyme inhibitor.

|  |  |  | dose-response line | | | | pre-treatment * acaricide dose effect | | | pre-treatment effect | | |  |  |
| --- | --- | --- | --- | --- | --- | --- | --- | --- | --- | --- | --- | --- | --- | --- |
| acaricide | pre-treatment | n | slope ± SE | intercept ± SE | X^2^ | df | dev. | df | adj. p^¤^ | dev. | df | adj. p^¤^ | | |
| tau-fluvalinate | control | 528 | 1.59 ± 0.18 | -2.06 ± 0.19 | 5 | 5 | - | - | - | - | - | - | | |
|  | coumaphos^a^ | 421 | 0.93 ± 0.28 | 0.10 ± 0.15 | 28 | 5 | 2.98 | 1,11 | 0.35 | 27.9 | 2,12 | <0.01 | | |
|  | fenpyroximate^a^ | 425 | 1.76 ± 0.25 | -0.67 ± 0.17 | 10 | 4 | 0.32 | 1,10 | 1.00 | 60.8 | 2,11 | <0.01 | | |
|  | amitraz^a^ | 419 | 1.61 ± 0.32 | -0.92 ± 0.22 | 31 | 7 | 0.00 | 1,13 | 1.00 | 18.9 | 2,14 | <0.01 | | |
|  | thymol^a^ | 564 | 1.35 ± 0.13 | -1.36 ± 0.13 | 8 | 7 | 1.11 | 1,13 | 1.00 | 14.2 | 2,14 | <0.01 | | |
|  | oxalic acid^a^ | 447 | 1.41 ± 0.13 | -1.20 ± 0.12 | 6 | 7 | 0.72 | 1,13 | 1.00 | 30.4 | 2,14 | <0.01 | | |
|  | pyraclostrobin^b^ | 304 | 1.96 ± 0.66 | -1.27 ± 0.48 | 33 | 4 | 0.43 | 1,10 | 1.00 | 10.5 | 2,11 | 0.02 | | |
|  | boscalid^b^ | 303 | 2.58 ± 0.51 | -2.74 ± 0.54 | 15 | 4 | 4.63 | 1,10 | 0.11 | 4.65 | 2,11 | 0.34 | | |
|  | pyr. + bosc.^b^ | 471 | 2.88 ± 0.47 | -2.23 ± 0.37 | 21 | 6 | 8.12 | 1,12 | <0.01 | 23.0 | 2,13 | <0.01 | | |
|  | chlorothalonil^b^ | 421 | 1.77 ± 0.38 | -1.52 ± 0.36 | 24 | 5 | 0.20 | 1,11 | 1.00 | 10.5 | 2,12 | 0.01 | | |
|  | prochloraz^b^ | 381 | 1.94 ± 0.32 | 3.87 ± 0.64 | 14 | 4 | 0.99 | 1,10 | 1.00 | 72.3 | 2,11 | <0.01 | | |
| coumaphos | control | 420 | 3.12 ± 0.67 | -4.66 ± 0.97 | 15 | 4 | - | - | - | - | - | - | | |
|  | tau-fluvalinate^a^ | 713 | 1.48 ± 0.13 | -1.20 ± 0.12 | 12 | 7 | 12.0 | 1,12 | <0.01 | 40.7 | 2,13 | <0.01 | | |
|  | fenpyroximate^a^ | 335 | 1.80 ± 0.17 | -1.11 ± 0.14 | 5 | 5 | 6.34 | 1,10 | 0.03 | 50.6 | 2,11 | <0.01 | | |
|  | amitraz^a^ | 358 | 1.70 ± 0.51 | -1.64 ± 0.58 | 32 | 4 | 2.78 | 1,9 | 0.44 | 10.9 | 2,10 | 0.02 | | |
|  | thymol^a^ | 241 | 4.51 ± 1.00 | -6.15 ± 1.40 | 9 | 3 | 1.39 | 1,8 | 1.00 | 2.46 | 2,9 | 1.00 | | |
|  | oxalic acid^a^ | 188 | 1.75 ± 0.25 | -2.05 ± 0.25 | 3 | 3 | 4.67 | 1,8 | 0.16 | 9.37 | 2,9 | 0.07 | | |
|  | boscalid^b^ | 204 | 3.23 ± 0.56 | -4.38 ± 0.78 | 5 | 3 | 0.02 | 1,8 | 1.00 | 1.87 | 2,9 | 0.37 | | |
|  | pyr. + bosc.^b^ | 203 | 4.52 ± 1.20 | -6.32 ± 1.70 | 13 | 3 | 1.20 | 1,8 | 1.00 | 1.26 | 2,9 | 1.00 | | |
|  | chlorothalonil ^b^ | 264 | 1.81 ± 0.52 | -2.21 ± 0.62 | 34 | 5 | 2.29 | 1,10 | 0.53 | 4.20 | 2,11 | 0.43 | | |
|  | prochloraz^b^ | 365 | 3.11 ± 0.27 | 1.12 ± 0.13 | 3 | 4 | 0.00 | 1,9 | 1.00 | 101 | 2,10 | <0.01 | | |
| fenpyroximate | control | 503 | 2.66 ± 0.55 | -2.19 ± 0.48 | 43 | 5 | - | - | - | - | - | - | | |
|  | tau-fluvalinate^a^ | 473 | 1.67 ± 0.39 | -1.24 ± 0.29 | 29 | 5 | 2.24 | 1,11 | 1.00 | 1.53 | 2,12 | 1.00 | | |
|  | coumaphos^a^ | 522 | 2.18 ± 0.19 | -0.67 ± 0.07 | 42 | 6 | 0.42 | 1,12 | 1.00 | 7.75 | 2,13 | 0.02 | | |
|  | amitraz^a^ | 211 | 4.07 ± 0.49 | -1.04 ± 0.14 | 1 | 3 | 1.38 | 1,9 | 1.00 | 10.3 | 2,10 | 0.03 | | |
|  | thymol^a^ | 387 | 2.26 ± 0.26 | -1.28 ± 0.17 | 11 | 6 | 0.44 | 1,12 | 1.00 | 3.74 | 2,13 | 0.42 | | |
|  | oxalic acid^a^ | 351 | 1.87 ± 0.46 | -0.33 ± 0.20 | 42 | 7 | 1.25 | 1,13 | 1.00 | 10.8 | 2,14 | <0.01 | | |
|  | pyraclostrobin^b^ | 385 | 2.27 ± 0.72 | -0.73 ± 0.36 | 44 | 5 | 0.19 | 1,11 | 1.00 | 7.52 | 2,12 | 0.04 | | |
|  | boscalid^b^ | 228 | 3.30 ± 0.96 | -2.48 ± 0.72 | 24 | 4 | 0.32 | 1,10 | 1.00 | 0.30 | 2,11 | 1.00 | | |
|  | pyr. + bosc.^b^ | 388 | 3.09 ± 0.32 | -1.54 ± 0.17 | 9 | 6 | 0.40 | 1,12 | 1.00 | 5.97 | 2,13 | 0.07 | | |
|  | chlorothalonil^b^ | 275 | 3.13 ± 0.32 | -2.52 ± 0.26 | 2 | 3 | 0.30 | 1,9 | 1.00 | 0.15 | 2,10 | 1.00 | | |
|  | prochloraz^b^ | 460 | 2.17 ± 0.28 | 1.30 ± 0.17 | 15 | 6 | 0.71 | 1,12 | 1.00 | 42.0 | 2,13 | <0.01 | | |
|  | DEM^c^ | 317 | 1.60 ± 0.17 | -1.03 ± 0.15 | 14 | 4 | 2.79 | 1,10 | 0.82 | 2.32 | 2,11 | 1.00 | | |
|  | DEF^c^ | 219 | 2.44 ± 0.74 | -0.25 ± 0.25 | 16 | 3 | 0.05 | 1,9 | 1.00 | 11.0 | 2,10 | 0.02 | | |
|  | PBO^c^ | 404 | 1.90 ± 0.46 | 1.07 ± 0.34 | 30 | 4 | 1.13 | 1,10 | 1.00 | 19.6 | 2,11 | <0.01 | | |
| amitraz | control | 552 | 2.23 ± 0.35 | -1.00 ± 0.24 | 26 | 7 | - | - | - | - | - | - | | |
|  | tau-fluvalinate^a^ | 402 | 3.10 ± 0.76 | -2.13 ± 0.60 | 31 | 4 | 1.52 | 1,12 | 1.00 | 3.49 | 2,13 | 0.49 | | |
|  | coumaphos^a^ | 357 | 2.45 ± 0.41 | -1.07 ± 0.25 | 14 | 5 | 0.15 | 1,13 | 1.00 | 0.11 | 2,14 | 1.00 | | |
|  | fenpyroximate^a^ | 386 | 2.25 ± 0.42 | -1.48 ± 0.34 | 12 | 4 | 0.00 | 1,12 | 1.00 | 3.35 | 2,13 | 0.51 | | |
|  | thymol^a^ | 342 | 2.78 ± 0.35 | -1.64 ± 0.20 | 5 | 3 | 0.92 | 1,11 | 1.00 | 2.37 | 2,12 | 1.00 | | |
|  | oxalic acid^a^ | 756 | 2.81 ± 0.78 | -3.27 ± 0.85 | 100 | 7 | 1.00 | 1,15 | 0.88 | 19.6 | 2,16 | <0.01 | | |
|  | pyraclostrobin^b^ | 457 | 1.96 ± 0.36 | -0.42 ± 0.20 | 28 | 6 | 0.32 | 1,14 | 1.00 | 2.72 | 2,15 | 0.77 | | |
|  | boscalid^b^ | 284 | 3.89 ± 0.81 | -2.66 ± 0.62 | 9 | 3 | 3.75 | 1,11 | 0.26 | 4.23 | 2,12 | 0.36 | | |
|  | pyr. + bosc.^b^ | 317 | 3.04 ± 0.28 | -1.85 ± 0.17 | 44 | 5 | 0.25 | 1,10 | 1.00 | 0.28 | 2,11 | 1.00 | | |
|  | chlorothalonil^b^ | 526 | 1.98 ± 0.60 | -1.04 ± 0.35 | 51 | 5 | 0.15 | 1,13 | 1.00 | 0.36 | 2,14 | 1.00 | | |
|  | prochloraz^b^ | 559 | 2.66 ± 0.57 | -1.05 ± 0.31 | 39 | 5 | 0.46 | 1,13 | 1.00 | 0.56 | 2,14 | 1.00 | | |
|  | DEM^c^ | 354 | 3.10 ± 0.86 | -1.12 ± 0.44 | 21 | 3 | 1.19 | 1,11 | 1.00 | 1.52 | 2,12 | 1.00 | | |
|  | DEF^c^ | 476 | 1.99 ± 0.23 | -0.67 ± 0.12 | 9 | 5 | 0.33 | 1,13 | 1.00 | 1.01 | 2,14 | 1.00 | | |
|  | PBO^c^ | 436 | 3.05 ± 0.93 | -1.17 ± 0.43 | 51 | 4 | 0.94 | 1,12 | 1.00 | 0.77 | 2,13 | 1.00 | | |
| thymol | control | 902 | 2.32 ± 0.27 | -4.04 ± 0.50 | 34 | 8 | - | - | - | - | - | - | | |
|  | tau-fluvalinate^a^ | 382 | 2.11 ± 0.29 | -2.55 ± 0.41 | 14 | 7 | 0.24 | 1,16 | 1.00 | 21.7 | 2,17 | <0.01 | | |
|  | coumaphos^a^ | 433 | 3.33 ± 0.87 | -4.35 ± 1.20 | 51 | 5 | 2.01 | 1,14 | 1.00 | 9.75 | 2,15 | <0.01 | | |
|  | fenpyroximate^a^ | 541 | 2.09 ± 0.27 | -3.22 ± 0.48 | 27 | 8 | 0.36 | 1,17 | 1.00 | 3.24 | 2,18 | 0.33 | | |
|  | amitraz^a^ | 331 | 3.83 ± 0.89 | -6.27 ± 1.50 | 30 | 5 | 3.60 | 1,14 | 0.01 | 3.51 | 2,15 | 0.37 | | |
|  | oxalic acid^a^ | 375 | 2.92 ± 0.42 | -4.34 ± 0.64 | 15 | 6 | 1.26 | 1,15 | 0.33 | 6.47 | 2,16 | 0.02 | | |
|  | pyraclostrobin^b^ | 488 | 1.44 ± 0.42 | -2.09 ± 0.72 | 49 | 6 | 3.25 | 1,15 | 0.17 | 4.02 | 2,16 | 0.18 | | |
|  | boscalid^b^ | 438 | 2.50 ± 0.38 | -4.18 ± 0.64 | 13 | 5 | 0.12 | 1,14 | 1.00 | 0.57 | 2,15 | 1.00 | | |
|  | pyr. + bosc.^b^ | 328 | 2.77 ± 0.71 | -4.17 ± 1.20 | 22 | 4 | 0.39 | 1,13 | 1.00 | 4.74 | 2,14 | 0.14 | | |
|  | chlorothalonil^b^ | 467 | 2.25 ± 0.33 | -3.32 ± 0.53 | 29 | 9 | 0.03 | 1,18 | 1.00 | 6.26 | 2,19 | 0.01 | | |
|  | prochloraz^b^ | 559 | 2.66 ± 0.20 | -1.05 ± 0.11 | 39 | 5 | 8.34 | 1,13 | <0.01 | 8.87 | 2,14 | 0.01 | | |
|  | DEM^c^ | 543 | 2.47 ± 0.44 | -4.46 ± 0.82 | 40 | 7 | 0.09 | 1,16 | 1.00 | 0.37 | 2,17 | 1.00 | | |
|  | DEF^c^ | 750 | 2.48 ± 0.48 | -3.83 ± 0.79 | 54 | 6 | 0.10 | 1,15 | 1.00 | 3.06 | 2,16 | 0.49 | | |
|  | PBO^c^ | 643 | 2.23 ± 0.41 | -3.36 ± 0.66 | 67 | 9 | 0.04 | 1,18 | 1.00 | 3.21 | 2,19 | 0.30 | | |

^¤^p-values adjusted for 73 pairwise comparisons with Holm-Bonferroni. Letters on pre-treatment names indicate class of compound: a=acaricide, b=fungicide, c=model enzyme inhibitor. Acetone control pre-treatments, to which all other pre-treatments are compared, are indicated with a dash.
