# Supplementary material for: Acaricide, Fungicide and Drug Interactions in Honey Bees (Apis mellifera)
Source: PLoS One. 2013 Jan 29;8(1):e54092. doi: 10.1371/journal.pone.0054092 (PMC3558502; doi:10.1371/journal.pone.0054092)
Supplement: Table S2 — Dose-response line parameters and pairwise comparisons for topical application of five acaricides following 24 h oral treatment with antimicrobial drugs. (DOCX) [file pone.0054092.s002.docx]

**Table S2.** Dose-response line parameters and pairwise comparisons for topical application of five acaricides following 24 h. oral treatment with antimicrobial drugs.

|  | |  |  | dose-response line | | | | pre-treatment * acaricide dose effect | | | pre-treatment effect | | |  |
| --- | --- | --- | --- | --- | --- | --- | --- | --- | --- | --- | --- | --- | --- | --- |
| acaricide | pre-treatment | | n | slope ± SE | intercept ± SE | X^2^ | df | dev. | df | adj. p^¤^ | dev. | df | adj. p^¤^ | |
| tau-fluvalinate. | control | | 1061 | 1.98 ± 0.14 | -1.91 ± 0.13 | 13 | 9 | - | - | - | - | - | - | |
|  | oxytetracycline | | 771 | 2.68 ± 0.21 | -2.48 ± 0.19 | 10 | 7 | 8.15 | 17 | <0.01 | 4.09 | 2,18 | 0.11 | |
|  | tylosin | | 700 | 2.32 ± 0.34 | -2.37 ± 0.32 | 22 | 6 | 1.09 | 16 | 1.00 | 1.69 | 2,17 | 1.00 | |
|  | fumagillin | | 757 | 1.99 ± 0.22 | -1.36 ± 0.16 | 30 | 10 | 0.00 | 20 | 1.00 | 12.4 | 2,21 | <0.01 | |
| coumaphos | control | | 283 | 4.27 ± 0.83 | -6.05 ± 1.1 | 9 | 3 | - | - | - | - | - | - | |
|  | oxytetracycline | | 609 | 2.47 ± 0.36 | -3.22 ± 0.46 | 18 | 5 | 4.04 | 9 | 1.00 | 4.45 | 2,10 | 0.45 | |
|  | tylosin | | 262 | 3.50 ± 0.72 | -4.93 ± 0.99 | 9 | 3 | 0.50 | 7 | 1.00 | 0.28 | 2,8 | 1.00 | |
|  | fumagillin | | 487 | 2.13 ± 0.28 | -3.24 ± 0.37 | 10 | 6 | 8.88 | 10 | 0.07 | 4.56 | 2,11 | 0.36 | |
| fenpyroximate | control | | 533 | 2.65 ± 0.24 | -1.35 ± 0.16 | 10 | 6 | - | - | - | - | - | - | |
|  | oxytetracycline | | 429 | 3.50 ± 0.37 | -2.36 ± 0.28 | 9 | 5 | 4.08 | 12 | 0.07 | 7.88 | 2,13 | 0.02 | |
|  | tylosin | | 558 | 2.60 ± 0.18 | -1.58 ± 0.14 | 5 | 7 | 0.04 | 14 | 1.00 | 3.18 | 2,15 | 0.49 | |
|  | fumagillin | | 713 | 2.78 ± 0.32 | -2.06 ± 0.27 | 20 | 6 | 0.10 | 13 | 1.00 | 9.73 | 2,14 | 0.01 | |
| amitraz | control | | 615 | 2.99 ± 0.38 | -2.21 ± 0.33 | 29 | 7 | - | - | - | - | - | - | |
|  | oxytetracycline | | 391 | 4.23 ± 0.54 | -2.38 ± 0.30 | 9 | 4 | 3.03 | 12 | 1.00 | 4.71 | 2,13 | 0.19 | |
|  | tylosin | | 670 | 3.29 ± 0.29 | -2.16 ± 0.22 | 15 | 7 | 0.40 | 15 | 1.00 | 1.46 | 2,16 | 1.00 | |
|  | fumagillin | | 404 | 3.75 ± 0.52 | -2.21 ± 0.34 | 16 | 5 | 1.40 | 13 | 1.00 | 3.29 | 2,14 | 0.49 | |
| thymol | control | | 411 | 4.32 ± 0.69 | -6.83 ± 1.1 | 11 | 3 | - | - | - | - | - | - | |
|  | oxytetracycline | | 413 | 3.58 ± 0.85 | -5.14 ± 1.3 | 31 | 4 | 0.44 | 8 | 1.00 | 1.92 | 2,9 | 1.00 | |
|  | tylosin | | 606 | 3.12 ± 0.75 | -4.71 ± 1.2 | 35 | 4 | 1.22 | 8 | 1.00 | 0.82 | 2,9 | 1.00 | |
|  | fumagillin | | 474 | 4.02 ± 0.41 | -5.64 ± 0.61 | 8 | 5 | 0.17 | 9 | 1.00 | 7.72 | 2,10 | 0.08 | |

^¤^p-values adjusted for 73 pairwise comparisons with Holm-Bonferroni. Acetone control pre-treatments, to which all other pre-treatments are compared, are indicated with a dash.
