# Supplementary material for: Acaricide, Fungicide and Drug Interactions in Honey Bees (Apis mellifera)
Source: PLoS One. 2013 Jan 29;8(1):e54092. doi: 10.1371/journal.pone.0054092 (PMC3558502; doi:10.1371/journal.pone.0054092)
Supplement: Table S3 — Dose-response line parameters and pairwise comparisons for tau-fluvalinate following treatment with sterol biosynthesis inhibiting fungicides. (DOCX) [file pone.0054092.s003.docx]

**Table S3.** Dose-response line parameters and pairwise comparisons for tau-fluvalinate following treatment with sterol biosynthesis inhibiting fungicides.

|  |  |  | dose-response line | | | | pre-treatment * acaricide dose effect | | | pre-treatment effect | | |  |
| --- | --- | --- | --- | --- | --- | --- | --- | --- | --- | --- | --- | --- | --- |
| pre-treatment | nmol / bee | n | slope ± SE | intercept ± SE | X^2^ | df | dev. | df | adj. p^¤^ | dev. | df | adj. p^¤^ | |
| control | - | 867 | 2.85 ± 0.26 | -2.72 ± 0.24 | 14 | 7 | - | - | - | - | - | - | |
| PBO | 0.1 | 787 | 1.62 ± 0.37 | -1.69 ± 0.34 | 74 | 9 | 5.90 | 1,17 | <0.01 | 2.96 | 2,18 | 0.06 | |
|  | 1 | 249 | 4.23 ± 0.55 | -3.92 ± 0.52 | 2.5 | 2 | 3.95 | 1,10 | 0.30 | 2.05 | 2,11 | 0.26 | |
|  | 10 | 240 | 1.69 ± 0.35 | -0.32 ± 0.11 | 6.5 | 5 | 6.08 | 1,13 | 0.02 | 51.8 | 2,14 | <0.01 | |
| prochloraz | 0.1 | 411 | 2.98 ± 0.26 | -3.11 ± 0.27 | 4.8 | 5 | 0.10 | 1,13 | 1.00 | 3.00 | 2,14 | 0.08 | |
|  | 1 | 513 | 2.17 ± 0.44 | -1.45 ± 0.31 | 36 | 7 | 1.84 | 1,15 | 1.00 | 12.8 | 2,16 | <0.01 | |
|  | 10 | 304 | 2.00 ± 0.51 | 1.32 ± 0.41 | 31 | 5 | 2.59 | 1,13 | 0.66 | 52.4 | 2,14 | <0.01 | |
| propiconazole | 0.1 | 369 | 3.24 ± 0.32 | -3.71 ± 0.36 | 4.4 | 4 | 0.75 | 1,12 | 1.00 | 11.8 | 2,13 | <0.01 | |
|  | 1 | 587 | 2.46 ± 0.36 | -1.90 ± 0.29 | 19 | 6 | 0.83 | 1,14 | 1.00 | 8.63 | 2,15 | <0.01 | |
|  | 10 | 305 | 3.37 ± 0.37 | 0.44 ± 0.10 | 6.0 | 5 | 1.13 | 1,13 | 1.00 | 113 | 2,14 | <0.01 | |
| fenbuconazole | 0.1 | 601 | 2.76 ± 0.34 | -2.82 ± 0.34 | 17 | 6 | 0.05 | 1,14 | 1.00 | 1.24 | 2,15 | 0.34 | |
|  | 1 | 438 | 2.50 ± 0.33 | -1.98 ± 0.28 | 12 | 6 | 0.72 | 1,14 | 1.00 | 7.40 | 2,15 | <0.01 | |
|  | 10 | 765 | 2.52 ± 0.24 | 0.66 ± 0.10 | 14 | 7 | 0.94 | 1,15 | 1.00 | 136 | 2,16 | <0.01 | |
| metconazole | 0.1 | 263 | 3.54 ± 0.74 | -3.91 ± 0.80 | 18 | 4 | 1.04 | 1,12 | 1.00 | 4.35 | 2,13 | 0.04 | |
|  | 1 | 469 | 2.41 ± 0.31 | -1.73 ± 0.24 | 13 | 7 | 1.20 | 1,15 | 1.00 | 16.3 | 2,16 | <0.01 | |
|  | 10 | 393 | 2.19 ± 0.22 | 0.76 ± 0.11 | 9.4 | 7 | 3.71 | 1,15 | 0.12 | 134 | 2,16 | <0.01 | |
| myclobutanil | 0.1 | 505 | 3.08 ± 0.35 | -3.43 ± 0.37 | 13 | 6 | 0.30 | 1,14 | 1.00 | 7.82 | 2,15 | <0.01 | |
|  | 1 | 643 | 1.92 ± 0.20 | -1.10 ± 0.12 | 16 | 9 | 8.49 | 1,17 | <0.01 | 39.4 | 2,18 | <0.01 | |
|  | 10 | 263 | 2.71 ± 0.28 | 2.49 ± 0.25 | 3.1 | 3 | 0.10 | 1,11 | 1.00 | 141 | 2,12 | <0.01 | |

^¤^ p-values adjusted for 18 pairwise comparisons with Holm-Bonferroni. Acetone control pre-treatments, to which all other pre-treatments are compared, are indicated with a dash.
