# Supplementary material for: Acaricide, Fungicide and Drug Interactions in Honey Bees (Apis mellifera)
Source: PLoS One. 2013 Jan 29;8(1):e54092. doi: 10.1371/journal.pone.0054092 (PMC3558502; doi:10.1371/journal.pone.0054092)
Supplement: Methods S1 — Sample R script for lethal dose determination and pairwise comparison of two dose-response curves. (DOC) [file pone.0054092.s004.doc]

Methods S1. Sample R script for lethal dose determination and pairwise comparison of two dose-response curves

## Get R from http://cran.r-project.org/

###########################################################

## Read in data

## Example data entered from the command line

Pesticide <- rep("thymol", 21)

Dose <- c(10, 20, 30, 50, 70, 100, 120, 150, 200, 300, 10, 13, 20, 30, 50, 70, 100, 120, 150, 200, 300)

Pretreat <- c(rep("acetone", 10), rep("chlorothalonil", 11))

Dead <- c(3, 17, 20, 44, 35, 93, 33, 71, 94, 105, 7, 4, 15, 33, 40, 32, 50, 32, 15, 33, 33)

Treated <- c(100, 82, 117, 97, 38, 123, 38, 87, 108, 112, 56, 39, 52, 53, 56, 36, 54, 32, 17, 35, 37)

data <- data.frame(Pesticide, Dose, Pretreat, Dead, Treated)

## Calculate log dose

data$ldose <- log10(data$Dose)

############################################

### LD function (copy and paste into R console)

###########################################

LD <- function(r, n, d, conf.level) {

## Set up a number series

p <- seq(1, 99, 1)

## r=number responding, n=number treated, d=dose (untransformed), confidence interval level,

mod <- glm(cbind(r, (n-r)) ~ log10(d), family = binomial(link=probit))

### Calculate heterogeneity correction to confidence intervals according to Finney, 1971, (p.

### 72, eq. 4.27; also called "h")

het = deviance(mod)/df.residual(mod)

if(het < 1){het = 1} ### Heterogeneity cannot be less than 1

## Extract slope and intercept

summary <- summary(mod, dispersion=het, cor = F)

intercept <- summary$coefficients[1]

interceptSE <- summary$coefficients[3]

slope <- summary$coefficients[2]

slopeSE <- summary$coefficients[4]

z.value <- summary$coefficients[6]

N <- sum(n)

## Intercept (alpha)

b0<-intercept

## Slope (beta)

b1<-slope

## Slope variance

vcov = summary(mod)$cov.unscaled

var.b0<-vcov[1,1]

## Intercept variance

var.b1<-vcov[2,2]

## Slope intercept covariance

cov.b0.b1<-vcov[1,2]

## Adjust alpha depending on heterogeneity (Finney, 1971, p. 76)

alpha<-1-conf.level

if(het > 1) {talpha <- -qt(alpha/2, df=df.residual(mod))} else {talpha <- -qnorm(alpha/2)}

## Calculate g (Finney, 1971, p 78, eq. 4.36)

## "With almost all good sets of data, g will be substantially smaller than 1.0 and

## seldom greater than 0.4."

g <- het * ((talpha^2 * var.b1)/b1^2)

## Calculate theta.hat for all LD levels based on probits in eta (Robertson et al., 2007, pg.

## 27; or "m" in Finney, 1971, p. 78)

eta = family(mod)$linkfun(p/100) #probit distribution curve

theta.hat <- (eta - b0)/b1

## Calculate correction of fiducial limits according to Fieller method (Finney, 1971,

## p. 78-79. eq. 4.35)

const1 <- (g/(1-g))*(theta.hat + cov.b0.b1/var.b1) # const1 <- (g/(1-g))*(theta.hat - cov.b0.b1/var.b1)

const2a <- var.b0 + 2*cov.b0.b1*theta.hat + var.b1*theta.hat^2 - g*(var.b0 - (cov.b0.b1^2/var.b1))

const2 <- talpha/((1-g)*b1) * sqrt(het * (const2a))

## Calculate the confidence intervals LCL=lower, UCL=upper (Finney, 1971, p. 78-79. eq. 4.35)

LCL <- (theta.hat + const1 - const2)

UCL <- (theta.hat + const1 + const2)

## Calculate variance for theta.hat (Robertson et al., 2007, pg. 27)

var.theta.hat <- (1/(theta.hat^2)) * ( var.b0 + 2*cov.b0.b1*theta.hat + var.b1*theta.hat^2 )

## Make a data frame from the data at all the different values

ECtable <- data.frame(

"p"=p,

"N"=N,

"EC"=10^theta.hat,

"LCL"=10^LCL,

"UCL"=10^UCL,

"slope"=slope,

"slopeSE"=slopeSE,

"intercept"=intercept,

"interceptSE"=interceptSE,

"z.value"=z.value,

"chisquare"=deviance(mod),

"df"=df.residual(mod),

"h"=het,

"g"=g,

"theta.hat"=theta.hat,

"var.theta.hat"=var.theta.hat)

## Select output level

return(ECtable)

}

#############################################################

## Use LD function to get LD50

## and associated statistics for each pretreatment

#############################################################

conf.level=0.95 ## Specify width of confidence intervals (0.95)

LD.level=50 ## Specify the lethal dose level (a whole number is required)

## Set up a number series for graphing

p <- seq(1, 99, 1)

## Subset

trt1 <- data[data$Pretreat == "acetone",] ## Subset pre-treatment 1 data

trt2 <- data[data$Pretreat == "chlorothalonil",] ## Subset pre-treatment 2 data

## Get LD50 and associated statistics for pretreatment 1

response.trt1 <- LD(trt1$Dead, trt1$Treated, trt1$Dose, conf.level)

print(paste(trt1$Pesticide[1], "-", trt1$Pretreat[1], " LD", LD.level, "= ", round(response.trt1[LD.level,"EC"],3), conf.level*100, "% CI= ",round(response.trt1[LD.level,"LCL"], 3),"-", round(response.trt1[LD.level,"UCL"], 3)))

print(response.trt1[LD.level,])

## Get LD50 and associated statistics for pretreatment 2

response.trt2 <- LD(trt2$Dead, trt2$Treated, trt2$Dose, conf.level)

print(paste(trt2$Pesticide[1], "-", trt2$Pretreat[1], " LD", LD.level, "= ", round(response.trt2[LD.level,"EC"],3), conf.level*100, "% CI= ",round(response.trt2[LD.level,"LCL"], 3),"-", round(response.trt2[LD.level,"UCL"], 3)))

print(response.trt2[LD.level,])

###################################################

## Test for “Pre-treatment * Dose Effect” or “Hypothesis of Parallelism” with correction for

## heterogeneity (Robertson et al., 2007, p.31 and p.53 )

####################################################

mod1 <- glm(cbind(Dead, Treated-Dead) ~ Pretreat + Pretreat:ldose -1, family = binomial(link=probit), data=data) # Fit individual lines for all treatments with varying slopes

mod2 <- glm(cbind(Dead, Treated-Dead) ~ Pretreat + ldose -1, family = binomial(link=probit), data=data)

het = deviance(mod1)/df.residual(mod1) #Heterogeneity for fitted model

df1= df.residual(mod1)

df2= df.residual(mod2)

tstat.par=( (deviance(mod2)-deviance(mod1)) / (het*(df2-df1)))

testp.par= 1-pf(tstat.par, df1, df2)

df1.par=df2-df1

df2.par=df2

parallelout <- paste("parallel deviance =", round(tstat.par, 3), " df1=", df2-df1, " df2=", df2, "p=", round(testp.par, 3))

print(parallelout)

####################################################

## Test for “Pre-treatment Effect” or “Hypothesis of Equality” with correction for heterogeneity

## (Robertson et al., 2007, p.31 and 53 )

#####################################################

mod1 <- glm(cbind(Dead, Treated-Dead) ~ Pretreat + Pretreat:ldose -1, family = binomial(link=probit), data=data) # Fit

mod3 <- glm(cbind(Dead, Treated-Dead) ~ ldose, family = binomial(link=probit), data=data)

het = deviance(mod1)/df.residual(mod1) #Heterogeneity for fitted model

df1= df.residual(mod1)

df3= df.residual(mod3)

tstat.eq=( (deviance(mod3)-deviance(mod1)) / (het*(df3-df1)))

testp.eq= 1-pf(tstat.eq, df1, df3)

df1.eq = df3-df1

df2.eq = df3

equalityout <- paste("equality deviance =", round(tstat.eq, 4), " df1=", df1.eq, " df2=", df2.eq, "p=", round(testp.eq, 6))

print(equalityout)

##################################################

## Plot lines on log-probit plot

##################################################

plot(c(min(data$Dose),max(data$Dose)), c(-2,2), type = "n", xlab = paste(data$Pesticide[1], " dose (mcg/bee)"), ylab = "probits", log = "x")

text(10^trt1$ldose, qnorm(trt1$Dead/trt1$Treated), labels = as.character("*"), col = 1)

text(10^trt2$ldose, qnorm(trt2$Dead/trt2$Treated), labels = as.character("+"), col = 2)

# Draw the lines, in the assigned color

## Acetone line, CIs, parallel line

ld <- seq(-6, 6, 0.1)

lines(10^ld, qnorm(predict(mod1,data.frame(ldose=ld, Pretreat=factor(rep(trt1$Pretreat[1],length(ld)))), type="response")), col=1) # acetone line no-parallel

lines(response.trt1$UCL, qnorm(p*0.01), col = 1, lty = 3) # Upper CI from LD output

lines(response.trt1$LCL, qnorm(p*0.01), col = 1, lty = 3) # Lower CI from LD output

lines(10^ld, qnorm(predict(mod2, data.frame(ldose = ld, Pretreat = factor(rep(trt1$Pretreat[1], length(ld)), levels = levels(data$Pretreat))), type = "response")), col = 3, lty=2) # acetone line parallel

## Pretreat line, CIs, parallel line

lines(10^ld, qnorm(predict(mod1,data.frame(ldose=ld, Pretreat=factor(rep(trt2$Pretreat[2],length(ld)))), type="response")), col=2) # compare line no-parallel

lines(response.trt2$UCL, qnorm(p*0.01), col = 2, lty = 3) # Upper CI acetone

lines(response.trt2$LCL, qnorm(p*0.01), col = 2, lty = 3) # Lower CI acetone

lines(10^ld, qnorm(predict(mod2, data.frame(ldose = ld, Pretreat = factor(rep(trt2$Pretreat[1], length(ld)), levels = levels(data$Pretreat))), type = "response")), col = 3, lty=2) # compare line parallel

## Equality line

lines(10^ld, qnorm(predict(mod3, data.frame(ldose = ld, Pretreat = factor(rep(trt2$Pretreat[1], length(ld)), levels = levels(data$Pretreat))), type = "response")), col = 4, lty=4) # compare line parallel

## Legend

legend("topleft", col = c(1,2,3,4), pch = c("*", "+", "", ""), lty= c(NA,NA,2,4), legend = c(paste(trt1$Pretreat[1], "=", round(mod1$coefficients[1], 3), "+", round(mod1$coefficients[3], 3), "x") , paste(trt2$Pretreat[1], "=", round(mod1$coefficients[2],3), "+", round(mod1$coefficients[4], 3), "x"), parallelout, equalityout))
